# FHL1 Reduces Dystrophy in Transgenic Mice Overexpressing *FSHD Muscular Dystrophy Region Gene 1 (FRG1)*

**DOI:** 10.1371/journal.pone.0117665

**Published:** 2015-02-19

**Authors:** Sandra J. Feeney, Meagan J. McGrath, Absorn Sriratana, Stefan M. Gehrig, Gordon S. Lynch, Colleen E. D’Arcy, John T. Price, Catriona A. McLean, Rossella Tupler, Christina A. Mitchell

**Affiliations:** 1 Department of Biochemistry & Molecular Biology, Monash University, Clayton, Victoria, 3800, Australia; 2 Basic and Clinical Myology Laboratory, Department of Physiology, The University of Melbourne, Victoria, 3010, Australia; 3 Centre for Chronic Disease Prevention and Management, College of Health and Biomedicine, Victoria University, Melbourne, Victoria, 8001, Australia; 4 Department of Anatomical Pathology, Alfred Hospital, Prahran, Victoria, 3004, Australia; 5 Department of Medicine, Central Clinical School, Monash University, Clayton, VIC, 3800, Australia; 6 Program in Gene Function and Expression, University of Massachusetts Medical School, Worcester, MA, 01655, United States of America; 7 Dipartimento di Scienze della Vita, Universita di Modena e Reggio Emilia, 41125, Modena, Italy; University of Minnesota Medical School, UNITED STATES

## Abstract

Facioscapulohumeral muscular dystrophy (FSHD) is an autosomal-dominant disease with no effective treatment. The genetic cause of FSHD is complex and the primary pathogenic insult underlying the muscle disease is unknown. Several disease candidate genes have been proposed including *DUX4* and *FRG1*. Expression analysis studies of FSHD report the deregulation of genes which mediate myoblast differentiation and fusion. Transgenic mice overexpressing FRG1 recapitulate the FSHD muscular dystrophy phenotype. Our current study selectively examines how increased expression of FRG1 may contribute to myoblast differentiation defects. We generated stable C2C12 cell lines overexpressing FRG1, which exhibited a myoblast fusion defect upon differentiation. To determine if myoblast fusion defects contribute to the *FRG1* mouse dystrophic phenotype, this strain was crossed with skeletal muscle specific *FHL1*-transgenic mice. We previously reported that FHL1 promotes myoblast fusion *in vitro* and *FHL1*-transgenic mice develop skeletal muscle hypertrophy. In the current study, *FRG1* mice overexpressing FHL1 showed an improvement in the dystrophic phenotype, including a reduced spinal kyphosis, increased muscle mass and myofiber size, and decreased muscle fibrosis. FHL1 expression in *FRG1* mice, did not alter satellite cell number or activation, but enhanced myoblast fusion. Primary myoblasts isolated from *FRG1* mice showed a myoblast fusion defect that was rescued by FHL1 expression. Therefore, increased FRG1 expression may contribute to a muscular dystrophy phenotype resembling FSHD by impairing myoblast fusion, a defect that can be rescued by enhanced myoblast fusion via expression of FHL1.

## Introduction

FSHD region gene 1 (FRG1) is an evolutionarily conserved protein [1], associated with the inherited muscle disease Facioscapulohumeral muscular dystrophy (FSHD) [2]. The role of FRG1 in skeletal muscle is not fully understood, however it has reported roles in mRNA splicing [2–4] and actin-bundling [5,6]. Maintenance of FRG1 expression levels are important for normal skeletal muscle. In *Xenopus laevis* both *FRG1* overexpression and morpholino-mediated inhibition result in muscle abnormalities [7].

FSHD is an autosomal-dominant inherited disease with a prevalence ranging from 1:14,000–20,000 [8–11] However, the frequency of FSHD can be underestimated due to the high degree of clinical variability and the large proportion of patients with only mild symptoms. A recent population study reported the incidence as high as ∼1:8,500 (12/100,000) [12] FSHD is characterized by the progressive wasting of muscles, frequently commencing with weakening of facial muscles, and eventually progressing to the pelvic-girdle muscles affecting the ability to walk. Individuals with the most prevalent form of FSHD (FSHD Type 1) have contractions of a 3.3kb macrosatellite repeat array, D4Z4, located in the subtelomeric region of chromosome 4 (4q35) [13]. The most widely accepted FSHD disease gene, *DUX4*, resides within each D4Z4 repeat and encodes the double-homeodomain transcription factor DUX4 [14]. Contractions of the D4Z4 repeat result in chromatin relaxation and de-repression of DUX4 expression [15]. Multiple DUX4-target genes have been identified [16–18] and their potential involvement in the pathogenesis of FSHD examined [19]. In zebrafish, expression of DUX4 results in muscle abnormalities [20], however, although mice carrying human FSHD D4Z4 arrays recapitulate the important epigenetic profiles for FSHD, they do not develop a muscular dystrophy phenotype [21]. A recently developed X-linked inducible-*DUX4*-transgenic mouse resulted in embryonic lethality in hemizygous male mice. Surviving male *DUX4*-transgenic mice exhibited muscle weakness (with the absence of dystrophic pathology) and reduced myoblast differentiation, but did not recapitulate a FSHD phenotype [22].

The *FRG1* gene maps approximately 100 kb proximal to the D4Z4 repeat array on chromosome 4 [23]. Individuals with larger deletions at the 4q35 locus including the D4Z4 repeat and loss of the *FRG1* gene, do not develop FSHD, supporting the potential involvement of FRG1 in this disease [24,25]. The molecular pathogenesis of FSHD is complex, contentious and not yet fully elucidated. Studies have suggested that FSHD may result from a complex inter-play of genetic and epigenetic events including the possible de-repression of a number of genes proximal to the D4Z4 repeat, including *FRG1* [26]. This lead to the hypothesis that FSHD may result from the collaborative effects of multiple genes including *FRG1*, *DUX4* and others (*FRG2* and *ANT1)*, which determines the final dystrophic phenotype. Many studies have addressed the deregulation of proximally located genes with inconclusive results. An initial study reported that *FRG1* expression was increased in FSHD muscle [27]. However, follow-up studies by different groups and using different techniques have failed to confirm the upregulation of FRG1 in FSHD affected muscle [28–32]. Despite the inconclusive results showing increased FRG1 expression in FSHD patient muscle, overexpression of FRG1 in animal models shows remarkable and reproducible similarity to the FSHD phenotype. *FRG1*-transgenic mice develop a muscular dystrophy phenotype that shares key histological and physiological features with FSHD including, abnormal curvature of the spine (kyphosis) and skeletal muscle atrophy involving characteristic FSHD affected muscles [2]. Gene expression profiling studies in muscle from *FRG1*-transgenic mice have also revealed a remarkably similar gene expression profile to FSHD patient muscle [33]. In *Xenopus laevis* the skeletal muscle abnormalities caused by FRG1 overexpression are accompanied by defects in vasculature [34], reminiscent of the retinal vasculopathy that can develop in FSHD [28]. Interestingly, a recent study presented a possible unifying model for the pathogenesis of FSHD by showing a direct interplay between two key FSHD disease genes, *DUX4* and *FRG1* [25]. The transcription factor DUX4 was shown to promote FRG1 expression by binding to putative enhancer elements within the human *FRG1* gene. FSHD is also a highly heterogeneous disease showing marked variability in age of onset, clinical severity and disease progression. This may be a consequence of the complex interplay between multiple genes coupled with significant variability in disease gene expression As such, it is imperative to characterize the individual functions of each candidate gene.

Recent studies have identified defects in muscle stem cells (satellite cells) [33] and myoblast fusion [35], which may contribute to disease pathogenesis in the dystrophic *FRG1* mouse model. Defects in myogenesis may also be an important pathological hallmark of FSHD as multiple expression analyses studies have documented the deregulation of genes that regulate myoblast differentiation and fusion [30,36–39]. Defects in myoblast fusion contribute to the pathogenesis of an increasing number of muscular dystrophies [40–42] compromising muscle growth and regeneration. In this regard, the identification of novel factors that enhance myoblast fusion may provide a therapeutic strategy for muscle diseases associated with myoblast fusion defects.

Here we addressed two key questions; does a myoblast fusion defect contribute to the pathogenesis of muscular dystrophy observed in *FRG1*-transgenic mice and if so, does enhanced myoblast fusion rescue the dystrophic phenotype. Four and a half LIM protein 1 (FHL1) is a protein that is highly expressed in skeletal muscle [43] and significant recent interest has focused on FHL1 and its role in the maintenance of healthy muscle since the discovery of *FHL1* mutations as the cause of human muscle disease [44–47]. We have previously reported wild type FHL1 promotes myoblast fusion *in vitro* and skeletal muscle-specific *FHL1*-transgenic mice exhibit enhanced muscle growth (hypertrophy) via activation of calcineurin/NFAT signaling [48]. Therefore, we investigate here whether FHL1 could enhance myoblast fusion in diseased muscle.

We report that FRG1 overexpression causes a defect in the myogenic pathway by impairing myoblast fusion. Critically, transgenic FHL1 expression in *FRG1* mice reduces muscle wasting and improves the dystrophic phenotype by driving enhanced myoblast fusion. These studies reveal that FRG1 overexpression contributes to dystrophy pathogenesis by impairing myoblast fusion and provides evidence that targeting enhanced myoblast fusion can reduce disease severity.

## Materials and Methods

### Ethics Statement

Ethics approval was obtained from the Monash Animal Research Platform Animal Ethics Committee (SOBSB/2008/61), Monash University, Melbourne, Australia, under the guidelines of the Australian Code of Practice for the Care and Use of Animals for Scientific Purposes.

### Animal strains and breeding

*FRG1*-transgenic mice and *FHL1*-transgenic mice were both generated using human *FRG1* or *FHL1* cDNA respectively. *FRG1*-transgenic mice [2] were obtained from Professor Rossella Tupler (University of Massachuetts Medical School, Worcester, MA) and maintained by breeding to C57BL6/J mice. Skeletal muscle-specific *FHL1*-transgenic mice were generated as previously described [48] using the human skeletal actin (HSA) promoter and were maintained by breeding to FVB/N wild type mice. *FRG1* mice over-expressing FHL1 were generated by breeding male *FRG1* mice with female *FHL1*-transgenic mice to generate wild type, *FRG1* and *FRG1*/*FHL1* littermates for analysis. Mice carrying *FRG1* and *FHL1* transgenes were identified by PCR amplification from genomic tail DNA as previously reported [2,48]. All mice colonies were maintained at the Monash Animal Research Platform, Monash University, Australia, with a 12-hour day/night cycle with access to food and water *ad libitum*. All experiments used male transgenic mice and sex-matched wild type littermates at 6 and 12 weeks of age.

### Antibodies

A list of antibodies used for experiments is included as S1 Table.

### Growth and stable transfection of C2C12 cells

The C2C12 mouse myoblast cell line [49] was purchased directly from ATCC (ATCC CRL1772) and was cultured in DMEM supplemented with 20% FBS and 2mM glutamine at 37°C/8%CO_2_. Stable cell lines were generated using the pIRESneo3 vector (Clontech). The pIRESneo3 vector was modified by subcloning a hemaglutinin (HA) epitope tag from the pCGN vector (Dr Tracey Wilson, WEHI, Australia) into the pIRESneo3 vector flanked by *NheI* and *AgeI* restriction sites (pIRESneo3-HA). The pIRES/FH-FRG1 construct was provided by Professor Rossella Tupler (University of Massachusetts Medical School, Worcester, MA), from which the *FRG1* cDNA was PCR amplified and subcloned into the *EcoR1* site of the modified pIRESneo3-HA vector. pIRESneo3-HA-FRG1 and pIRESneo3-HA (control) plasmids were transfected into C2C12 myoblasts using lipofectamine 2000 (Invitrogen) according to manufacturer’s instructions. Single, stably transfected C2C12 clones were isolated by sequential plating followed by selection in growth media containing 1 mg/ml G418 for up to 20 days, with selection media changed every 2–3 days. Several pIRESneo3-HA-FRG1 and pIRESneo3-HA clones were selected and amplified for further analysis. To confirm overexpression of HA-FRG1, cell lysates were prepared using Tris Saline pH 7.4 and 1% Triton X-100 and immunoblotted with HA (Covance, MMS-101R, 1:5000) and FRG1 (Abcam, 55024, 1:500)-specific antibodies. Lysates from C2C12 myoblasts transiently expressing HA-FRG1 (pCGN-FRG1) were used as a positive control.

### RNA extraction and qRT-PCR analysis

Total RNA was prepared from 80% confluent proliferating or differentiating mouse myoblasts using the Isolate RNA Mini Kit (Bioline) and total RNA from muscle tissue prepared using the RNeasy Fibrous Mini Kit (Qiagen) as per manufacturer’s instructions. 50ng RNA was used for first-strand cDNA synthesis with Affinity Script QPCR cDNA Synthesis Kit (Stratagene). Quantitative RT-PCR (qRT-PCR) analysis was performed on the Corbett Rotorgene 3000 (Qiagen) using Brilliant II SYBR Green QPCR Mastermix (Stratagene) and Quantitect Primer Assay for human FRG1 (Qiagen, Hs_FRG1_1_SG), human FHL1 (Qiagen, HS_FHL1_1_SG), mouse Gapdh (Qiagen, Mm_Gapdh_3_SG), mouse Pax7 (Qiagen, Mm_Pax7_1_SG), mouse myod (forward 5’-GCCCGCGCTCCAACTGCTCTGAT- 3’, reverse 5’ -CCTACGGTGGTGCGCCCTCTGC- 3’) [50], mouse myogenin (forward 5’ –GGGCCCCTGGAAGAAAAG- 3’, reverse 5’—AGGAGGCGCTGTGGGAGT-3’) [50], mouse suv4–20h1 (forward 5’ TCGCAGTCGCTAAATTCCTT 3’, reverse 5’ CGACCAGTTGACACAAACTTAC 3’)[35] and mouse Eid3 (forward 5’ AGTTCCTGGTTTTGGCCTCT 3’, reverse 5’ TCGCAGTCGCTAAATTCCTT 3’) [35]. Relative mRNA for each amplicon was quantified after normalization against GAPDH using the comparative 2^-∆∆Ct^ method [51]. For all samples the results are presented as the fold change over the baseline values from control samples (wild type muscle or vector control cells) as is the standard approach for the 2^-∆∆Ct^ method. Each amplicon was analyzed in triplicate in a 72 well rotor in three independent experiments.

### C2C12 differentiation and immunofluorescence staining

C2C12 cells stably transfected with HA-vector or HA-FRG1 were plated onto fibronectin-coated coverslips (5μg/ml; Sigma) at 1x10^5^ cells per well (6-well dish for immunofluorescence) or onto fibronectin-coated 60mm dishes at 2.2x10^5^ cells per dish (for Western blot analyses). To induce differentiation, 80% confluent cells were washed with PBS and switched to DMEM supplemented with 2% horse serum, 2mM glutamine, and maintained for 0–96 hours at 37°C/8%CO_2_. At 24 hour intervals post differentiation, cells were fixed in 4% formaldehyde for myosin heavy chain immunofluorescence analysis as described [52] and visualized using a Nikon C1 confocal inverted microscope, or lysates prepared for western blotting analysis. For Western blot analyses, Triton X-100–soluble lysates were prepared as described [52]. Protein concentration was measured using a DC protein assay reagent (Bio-Rad Laboratories), and 25μg lysates were separated by SDS-PAGE and immunoblotted with antibodies specific for myogenin [48] (Santa Cruz Biotechnology Cat# sc-12732, RRID:AB_627980, 1:500); MHC [53] (Developmental Studies Hybridoma Bank Cat# MF 20, RRID:AB_2147781, 1/500) and β-tubulin [54] (Life Technologies Cat# 480011, RRID:AB_10375603, 1:5000). Ponceau red staining of membranes was used to confirm equal protein loading. Western blot films were scanned and band signal intensities determined using MacBiophotonics ImageJ v1.43m software. Protein expression was corrected for protein loading by standardizing to the corresponding β-tubulin values (protein loading control) and expressed as the fold difference to control (0 time point) cells. Each experiment was performed in triplicate.

### Mouse primary myoblasts

Primary myoblasts were derived from the hind limb muscles of mice post-natal day 1 and isolated using collagenase/dispase tissue digestion as described [55]. Primary myoblasts were cultured in growth media on 0.1% collagen (Sigma) coated plates at 37°C/8%CO_2_. The purity of all primary myoblast populations was confirmed by immunostaining for desmin. Myoblast cultures with >90% (range 89–95%) purity were used. For differentiation experiments, cells were plated at 5x10^4^cell per well in 24 well plates coated with fibronectin (5μg/ml; Sigma). Differentiation was induced after 2 hours plating by washing with PBS and switching to DMEM supplemented with 5% horse serum (Invitrogen), 1% insulin-transferrin-selenium-A solution and penicillin/streptomycin. Cultures were differentiated for 48 and 96 hours.

Differentiated primary myoblast cultures were fixed and permeabilized in 4% formaldehyde/0.01% triton X-100 for immunofluorescence analysis and co-stained for MHC (to identify myotubes) and nuclei (DAPI) as described [52]. Myoblast differentiation was visualized using a Leica AF 6000 LX optical microscope and assessed by quantifying the nuclear fusion index (proportion of nuclei localized within MHC-positive myotubes. Each experiment was performed in triplicate. For all cell counts, a minimum of five—ten random fields were scanned for each slide to ensure that ≥ 100 cells were scored for each replicate. Cell counts were quantified using MacBiophotonics ImageJ v1.43m software (National Institutes of Health, USA).

### Skeletal muscle lysates and Immunoblotting

The tibialis anterior, quadriceps, triceps and trapezius muscles were dissected, minced, and homogenized on ice for 5 × 30 s (Tissue Rupter, Qiagen) in 5 times the weight/volume of 1% NP-40, Tris-HCl buffer, pH 8, then extracted for 1 h at 4°C. Lysates were centrifuged at 1,000g for 5 minutes. Protein concentration was determined on the soluble fraction using a DC protein assay kit (Biorad), and 10μg of soluble protein was separated by SDS-PAGE and immunoblotted with antibodies specific for HA [48] (Covance Research Products Inc Cat# MMS-101R-500, RRID:AB_10063630, 1:5000), FRG1 (Abcam Cat# ab55024, RRID:AB_941653, 1:500), FHL1 [56] (Abcam Cat# ab23937, RRID:AB_732361, 1/1000), Eid3 [57] (Santa Cruz Biotechnology Cat# sc-167738, 1/500) and β-tubulin. Band signal intensities were determined as described above. Ponceau red staining of membranes was used to confirm equal protein loading.

### Skeletal muscle histology

Muscles were snap frozen in isopentane cooled in liquid nitrogen. Serial 6μm—10μm transverse cryosections of muscle were fixed and stained with Harris Haematoxlin and Eosin (H&E) for morphological analysis, Masson’s Trichrome for fibrosis staining and Oil Red O for fat staining. All stains were performed using standard techniques. Sections stained with H&E and Masson’s Trichrome were mounted with a glass coverslip using DPX (Grail Scientific). Sections stained with Oil Red O were mounted with a glass coverslip and aquatex (aqueous mounting medium; Grail Scientific). All muscle histology was viewed using an Olympus AX70 Provis fitted with an Olympus DP70 color camera and captured using AnalySiS 5 software.

All quantitative analysis of muscle was performed using MacBiophotonics ImageJ v1.43m software. Prior to analysis, a spatial calibration was performed on Image J to set the image scale. For quantification of the area of fibrosis (Masson’s Trichrome) and fat deposition (Oil Red O), a minimum threshold was first determined from analysis of wild type muscle sections by performing an automated threshold to include only the fibrosis- and fat-stained areas within normal healthy muscle. This minimum threshold was maintained for analysis of *FRG1* and *FRG1*/FHL1 muscle sections. The area occupied by fibrosis or fat deposition in muscle sections was determined from ten fibrosis- and fat-stained sections and expressed as the mean percentage area. Data represents the average of 4–6 mice per genotype for determination of fibrosis and 3–4 mice per genotype for determination of fat.

Muscle fiber diameter and centralized nuclei were analyzed using 8 μm transverse H&E stained muscle cryosections. Fiber Diameters were determined by measuring the minimal ferret’s diameter [58] using MacBiophotonics Image J. Between 500–1000 fibers were measured per muscle. Data represent the average of 10 sections from 3–5 mice per genotype. Histograms of fiber diameters were plotted and mean fiber diameter graphed and analyzed using GraphPad Prism 5 software. The total numbers of muscle fibers with centralized nuclei were counted from 500–1000 fibers per muscle. Data represent the mean from 3 wild type, 4 *FRG1* and 4 *FRG1*/*FHL1* mice.

### Skeletal muscle immunostaining

Immunofluorescence staining for Pax7 [59] (Developmental Studies Hybridoma Bank Cat# pax7, RRID:AB_528428, 1/50), and DAPI was performed on 10μm transverse cryosections using the Vector M.O.M Immunodetection kit (Vector laboratories) for tissue sections as per manufacturer’s instructions. Co-staining for Pax7 and MyoD (Cell Signaling Technology, Cat# 13812S, 1/400) was achieved by double immunofluorescent labeling. Sections were first stained with Pax7 as described above, then, stained overnight with rabbit anti-MyoD, washed in PBS and incubated with goat anti-rabbit Alexa Fluor (1:400; Molecular probes) for 2 hours at room temperature. Sections were washed in PBS and coverslips mounted using fluoromount-G mounting media (Emgrid Australia, Cat# 17984–25). Images of Pax7^+^/MyoD^-^, Pax7^+^/MyoD^+^ and Pax7^-^/MyoD^+^ stained cells were captured using an Olympus AX70 Provis fitted with an Olympus DP70 color camera and AnalySiS 5 software. All analyses were manually performed using Image J. The total number of Pax7^+^/MyoD^-^, Pax7^+^/MyoD^+^or Pax7^-^/MyoD^+^ cells from transverse sections was determined from 4–5 consecutive fields at x200 magnification for each muscle section. Data presented as cell number per 100 myofibers.

For the determination of nuclei number per mm myofiber length [60], 10μm longitudinal cryosections from triceps muscle were fixed in 4% paraformaldehyde, washed in PBS and permeabilised/blocked (10% horse serum, 1% BSA and 0.1% triton X-100 in PBS) for 1 hour at room temperature. Sections were stained overnight with rabbit anti-dystrophin (Abcam Cat# ab15277, RRID:AB_301813, 1:400) [61], washed in PBS and incubated with goat anti-rabbit Alexa Fluor 594 and DAPI (1:100) for 1 hour at room temperature. Sections were washed in PBS and coverslips mounted using fluoromount mounting media.

Images of Dystrophin/DAPI stained longitudinal sections were captured using an Olympus BX-51 microscope using dotSlide Software. All analyses were manually performed using Imaging Software CellSens v1.6. The number of nuclei from longitudinal sections was determined from 20–40 myofibers [62] at x200 magnification for each muscle section. Data presented as the number of nuclei per mm of myofiber length. Data represent the mean ± SEM from 3–4 wild type, 3–4 *FRG1* and 3–4 *FRG1/FHL1* mice.

### Analysis of muscle function

All procedures were approved by the Animal Experimentation Ethics Committee of The University of Melbourne (1112223) and conformed to the Guidelines for the Care and Use of Experimental Animals described by the National Health and Medical Research Council (Australia). Mice were housed in the Biological Research Facility at The University of Melbourne under a 12-hour light/dark cycle. *In situ* muscle function on mice was performed under sodium pentobarbitone anesthesia (Nembutal, 120 mg/kg), and all efforts were made to minimize suffering.

The methods for measuring contractile function of mouse tibialis anterior (TA) muscles *in situ* have been described in detail elsewhere [63]. Briefly, TA muscles were stimulated by supramaximal 0.2 ms square wave pulses of 300 ms duration, delivered via two wire electrodes adjacent to the peroneal nerve. Optimum muscle length (L_o_) was determined from maximum isometric twitch force (P_t_), and maximum isometric tetanic force (P_o_) was recorded from the plateau of a complete frequency-force relationship. Specific force (sP_o)_ was determined by normalizing P_o_ to muscle cross-sectional area (CSA) and expressed in kN/m^2^. The protocol for assessment of muscle fatigability was assessed using a standard fatigue protocol. Muscles were stimulated maximally once every 2 s for 4 minutes, with maximum force reported every minute. Muscles were trimmed of tendons and any adhering non-muscle tissue, blotted on filter paper and weighed on an analytical balance.

### Statistical analysis

Data were graphed and analyzed using GraphPad Prism 5. Results were reported as mean ± SEM for the total number of observations, where n equals the number of mice or experiments used. Two tailed Student’s *t* tests and two-way ANOVA with Tukey’s multiple comparisons test were used to evaluate statistical significance and calculate *P* values with threshold values as described in the results or figure legends. *P* values of <0.05 were considered as statistically significant.

## Results

### C2C12 myoblasts over-expressing FRG1 exhibit a myoblast fusion defect

**Fig 1:**
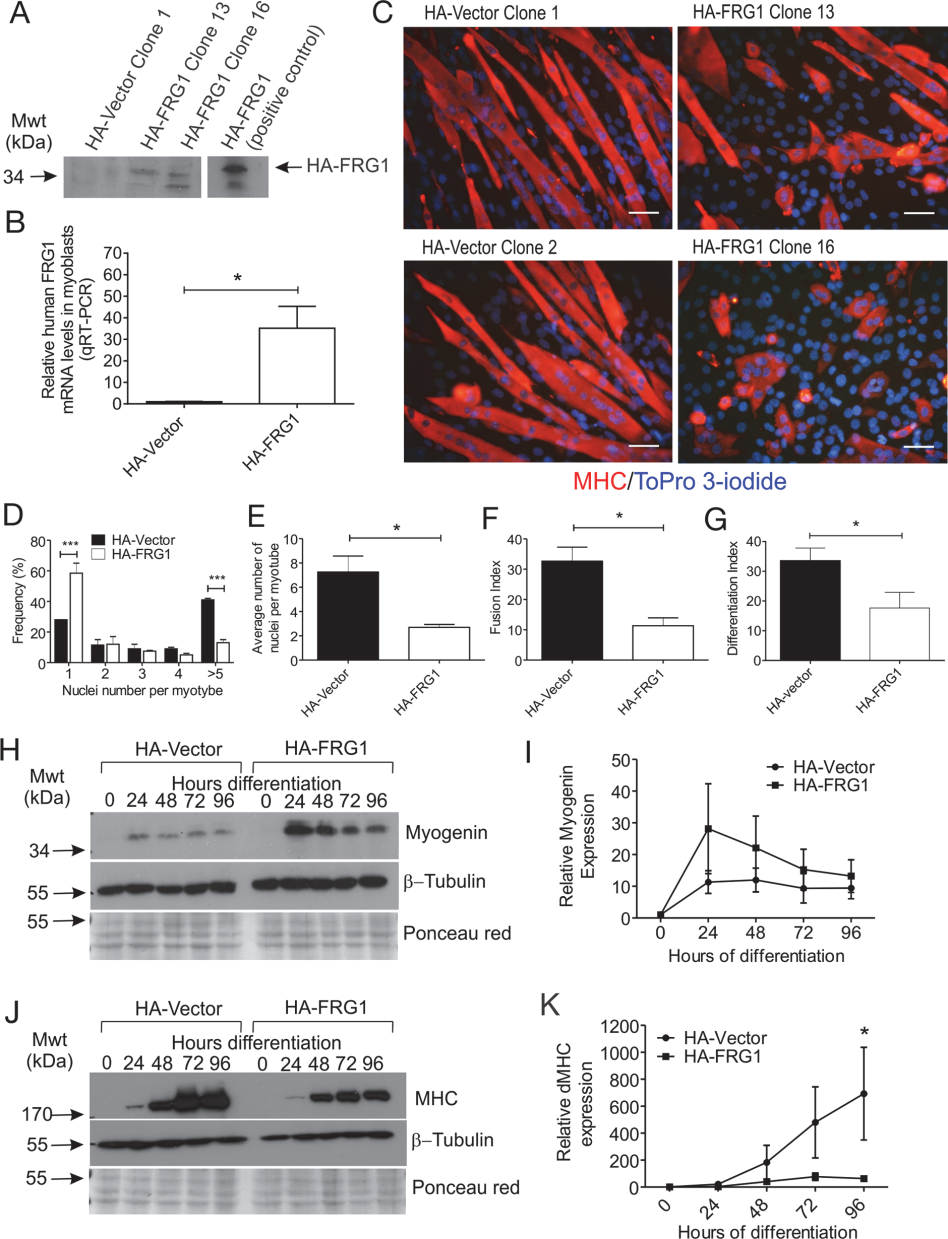
C2C12 myoblasts overexpressing FRG1 exhibit a fusion defect. (A) Immunoblot analysis of FRG1 expression in C2C12 myoblasts expressing HA-vector or HA-FRG1. HA-tagged FRG1 was detected using a HA-specific antibody. Clones HA-FRG1 13 and HA-FRG1 16 were selected for further analysis. Positive control represents HA-FRG1 transfected COS1 cells. (B) qRT-PCR analysis of FRG1 mRNA levels in undifferentiated HA-FRG1 myoblasts relative to HA-vector control myoblasts. Data represent the mean +/- SEM from n = 3 independent experiments; *p<0.05. (C) Representative images of C2C12 myoblasts expressing either HA-vector control or HA-FRG1 as indicated, following 96 hours differentiation and stained with the differentiation marker MHC (red) and ToPro 3-iodide to detect nuclei (blue). (D-F) Several parameters were quantified to assess the efficiency of myoblast differentiation; (D) Frequency of MHC-positive myoblasts and myotubes containing 1, 2, 3, 4 or ≥5 nuclei. Data represent the mean ± SEM from n = 3 independent experiments;***p<0.0005 determined by two-way ANOVA with Tukey’s multiple comparisons test; (E) Average number of nuclei per myotube; (F) Fusion index; the percentage of total nuclei localized within MHC-positive myotubes; (G) Differentiation index; the percentage of total nuclei localized within MHC-positive cells (myocytes and myotubes); (H-I) Relative myogenin and (J-K) MHC expression in HA-vector *versus* HA-FRG1 expressing myoblasts during 0–96 hours differentiation. Immunoblotting for β-tubulin and staining membranes with ponceau red were used as a loading control. Myogenin and MHC expression were quantified using densitometry. Data for (B), (E-F), (I) and (K) represent the mean ± SEM from n = 3 independent experiments; *p< 0.05 determined by two-tailed Student’s T-test. Scale bars = 50μm.

We selectively examined the effect of FRG1 overexpression on myogenesis by generating several C2C12 myoblast cell lines stably over-expressing HA-tagged FRG1. Immunoblot analysis using a HA-specific antibody confirmed expression of HA-FRG1 in two independent clones (Clones 13 and 16), which were selected for further analysis (Fig. 1A). HA-vector stably transfected cells were used as a control. QRT-PCR analysis demonstrated increased FRG1 mRNA in C2C12 myoblasts stably expressing HA-FRG1 relative to control HA-vector myoblasts (Fig. 1B). Myoblast fusion was evaluated by co-staining cultures for a myotube-specific marker myosin heavy chain (MHC), and ToPro 3-iodide to detect nuclei (as previously) [52]. Control myoblasts fused to form long, multinucleated MHC-positive myotubes that contained an average of 7 or more nuclei after 96 hours differentiation (Fig. 1C-E). In contrast, most C2C12 myoblasts over-expressing FRG1 remained as mononucleated myoblasts (Fig. 1C-D), or fused to form short rounded myotubes containing small numbers of nuclei (Fig. 1C-E). This myoblast fusion defect was observed for both HA-FRG1 clones. Analysis of all HA-vector clones *versus* all HA-FRG1 clones confirmed a 3-fold decrease in myoblast fusion with FRG1 overexpression (Fig. 1F) which was accompanied by an overall decrease in the proportion of MHC+ cells (Fig. 1G).

To further define the myoblast fusion defect observed in FRG1-expressing myoblasts, expression of the myogenic regulatory transcription factor myogenin, which initiates differentiation and is typically induced within 24 hours following the induction of C2C12 myoblast differentiation [48], was examined. Expression of its downstream target MHC was also evaluated (Fig. 1H-K). Surprisingly, despite a myoblast fusion defect, a trend towards increased myogenin expression was observed in FRG1-expressing myoblasts undergoing differentiation, relative to control myoblasts (Fig. 1H-I). Myogenin mRNA is also increased in the muscles of *FRG1*-transgenic mice [4], an observation consistent with our study showing increased myogenin protein expression in cultured myoblasts overexpressing FRG1. Analysis of MHC expression revealed a significant reduction in FRG1-overexpressing myoblasts (Fig. 1J-K), consistent with our immunofluorescence experiments showing an overall decrease in the proportion of MHC+ cells in FRG1 cultures (Fig. 1G). Therefore our data suggests that FRG1 overexpression does not inhibit initiation of the differentiation program, as shown by the expression of myogenin but, rather impairs later events including MHC expression and myoblast fusion.

### FHL1 reduces muscle wasting in the *FRG1* mouse

Our *in vitro* data revealed that overexpression of FRG1 in C2C12 mouse myoblasts results in a fusion defect. Therefore, to provide proof of principle that FRG1 can impair myoblast fusion leading to muscular dystrophy, we investigated if co-expression of an agent that promotes myoblast fusion could rescue the dystrophic phenotype of *FRG1* mice. We have previously reported that the LIM-only protein, FHL1, promotes myoblast fusion *in vitro* and skeletal muscle hypertrophy *in vivo* by enhancing NFAT transcriptional activity [48]. To determine if FHL1 expression can reduce muscle wasting in *FRG1* mice, we crossed our skeletal muscle-specific *FHL1*-transgenic mice [48] with the dystrophic *FRG1* mouse model [2]. A previous study has demonstrated that the effect of FRG1 overexpression on muscle is dose-dependent [2]. This was shown by generating *FRG1*-low, *FRG1*-med and *FRG1*-high transgenic mice, which express FRG1 at different levels. In the current study we use the *FRG1*-high line, which exhibits the most severe dystrophic phenotype.

**Fig 2:**
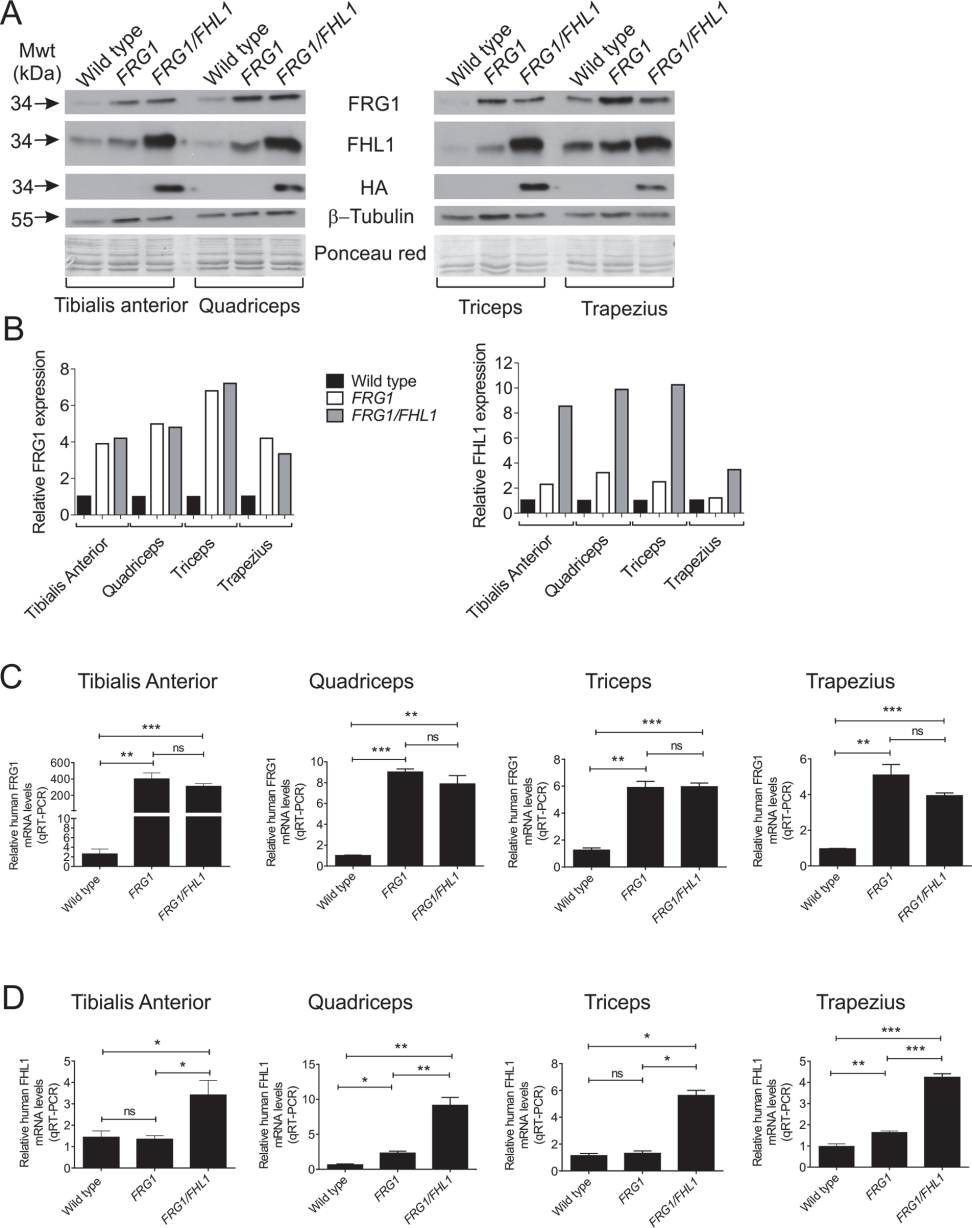
Generation of *FRG1* and *FRG1/FHL1* mice. (A) Immunoblot analysis of protein expression in the tibialis anterior, quadriceps, triceps and trapezius muscles from wild type, *FRG1* and *FRG1*/*FHL1* mice. HA-tagged FHL1 was detected using a HA-specific antibody; β-tubulin immunoblotting and ponceau red staining of membranes were used as loading controls. (B) Relative FRG1 and FHL1 expression levels in the tibialis anterior, quadriceps, triceps and trapezius muscles from wild type, *FRG1* and *FRG1*/*FHL1* mice. Protein expression was quantified using densitometry. Quantitative RT-PCR analysis of FRG1 (C) and FHL1 (D) mRNA in muscles from wild type, *FRG1* and *FRG1/FHL1* mice. Data represent the mean from n≥4 mice/genotype; *p<0.05, **p<0.005, ***p<0.001 determined by two-tailed student’s T-test.

Different muscles in *FRG1* mice are affected to varying extents and show pathological changes [2] in a distribution similar to FSHD [64]. Muscles affected in order of severity include the trapezius, vastus lateralis (quadriceps), triceps and the tibialis anterior [2]. Immunoblot analysis of these affected muscles confirmed increased FRG1 protein expression in *FRG1* and *FRG1*/*FHL1* mice relative to wild type littermates (Fig. 2A-B). The expression of transgenic HA-tagged FHL1 in *FRG1*/*FHL1* mouse muscles was detected by immunoblotting with a HA-specific antibody. Increased FHL1 protein expression was further confirmed using a FHL1 antibody, which detects both the endogenous and transgene-derived FHL1, and revealed a modest 4–5 fold increase in FHL1 expression in *FRG1*/*FHL1* muscle (Fig. 2B). QRT-PCR analysis using human-specific primers (to distinguish transgene-derived human transcripts from endogenous murine transcripts) confirmed a ∼5–9-fold increase in FRG1 mRNA across various muscles in *FRG1* and *FRG1/FHL1* mice (Fig. 2C). FHL1 mRNA was increased 4–10 fold in *FRG1/FHL1* muscles (Fig. 2D).

**Fig 3:**
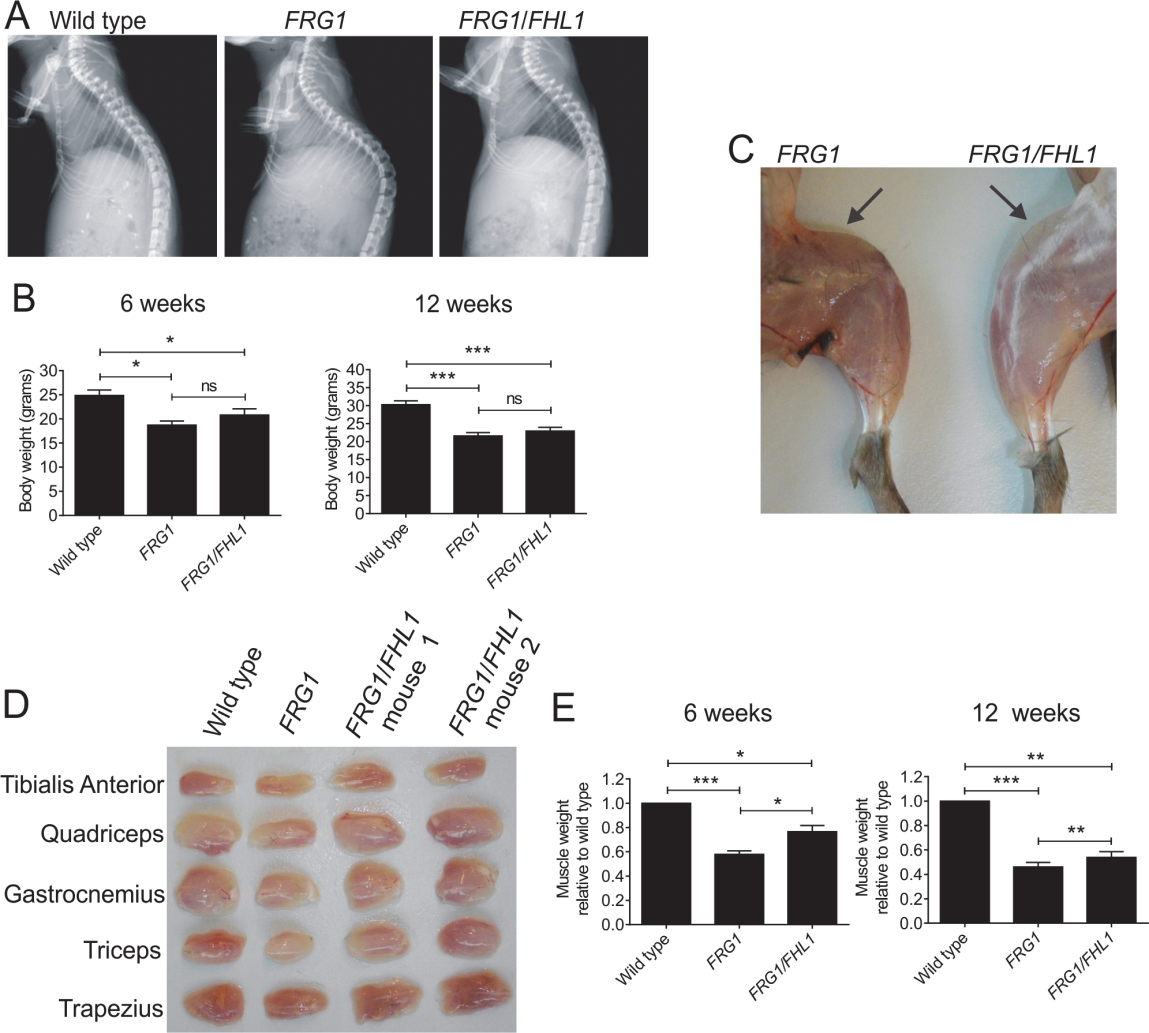
FHL1 reduces muscle wasting in dystrophic *FRG1* mice. (A) Representative X-ray images of the spine of mice from the indicated genotypes. (B) Whole body weight in 6-week-old mice; wild-type (n = 10); *FRG1* (n = 6); *FRG1*/*FHL1* (n = 9); and 12-week-old mice; wild-type (n = 11); *FRG1* (n = 12); *FRG1/FHL1* (n = 14). (C) Representative image of skinned hind limbs from *FRG1* and *FRG1*/*FHL1* mice. Arrows point to the quadriceps muscle to show the difference in muscle mass between the *FRG1* and *FRG1*/*FHL1* mice. (D) Representative images of various muscles dissected from wild type, *FRG1* and FRG1/*FHL1* mice. (E) Relative muscle weight from 6-week-old mice and 12-week-old mice. Data represents an average of the combined weights from 4 muscle groups (n = 6–10 mice per genotype for the tibialis anterior, quadriceps, triceps and trapezius) and is expressed relative to wild type muscle weight. Data represent the mean ± SEM; *p< 0.05; **p<0.005; ***p<0.0005 determined by two-tailed Student’s T-test.

Dystrophy in *FRG1* mice is characterized by progressive muscle wasting accompanied by spinal kyphosis (abnormal outward curvature of the spine), caused by muscle weakness [2]. X-ray images of representative 6-week-old *FRG1* mice confirmed the presence of kyphosis which was absent from wild type mice (Fig. 3A). Expression of FHL1 was sufficient to reduce the dystrophic phenotype of *FRG1* mice resulting in normal curvature of the spine, thus supporting the hypothesis that FHL1 expression can alleviate the reduced muscular support of the spine. Analysis of whole body weight revealed a significant reduction in *FRG1* relative to wild type mice (Fig. 3B), an effect previously shown to be caused by reduced muscle mass and not due to reduced caloric intake [2]. A trend towards increased body weight was observed in *FRG1/FHL1* mice relative to *FRG1* mice aged 6 weeks (but not at 12 weeks), but this difference was not statistically significant (Fig. 3B). However, examination of various muscles from mice at 6 weeks of age revealed FHL1 overexpression was sufficient to increase muscle mass in *FRG1* mice (Fig. 3C-E; S2 and S3 Tables). The weights of four affected muscle groups (tibialis anterior, quadriceps, triceps and trapezius) from *FRG1* mice showed a 40% reduction in muscle weight relative to wild type mice at 6 weeks of age (Fig. 3E). Significantly, a 33% increase in muscle mass in *FRG1*/*FHL1* mice was observed relative to *FRG1* littermates (aged 6 weeks) (Fig. 3E and S2 Table), which was sustained, albeit at lower levels (19% increase), in adult *FRG1*/*FHL1* mice aged 12 weeks (Fig. 3E and S3 Table). Therefore FHL1 promotes increased muscle mass in *FRG1* mice. This does not translate to an overall significant increase in body weight in *FRG1/FHL1* mice due to the small contribution of these muscles to overall body weight (∼1–2%). Collectively, this data provides evidence that we have achieved increased FHL1 expression in key affected muscles in the dystrophic *FRG1* mouse, and demonstrated that FHL1 expression is sufficient to reduce the severity of the dystrophic *FRG1* phenotype including amelioration of muscle wasting and spinal kyphosis.

### FHL1 improves muscle pathology in *FRG1* mice

**Fig 4:**
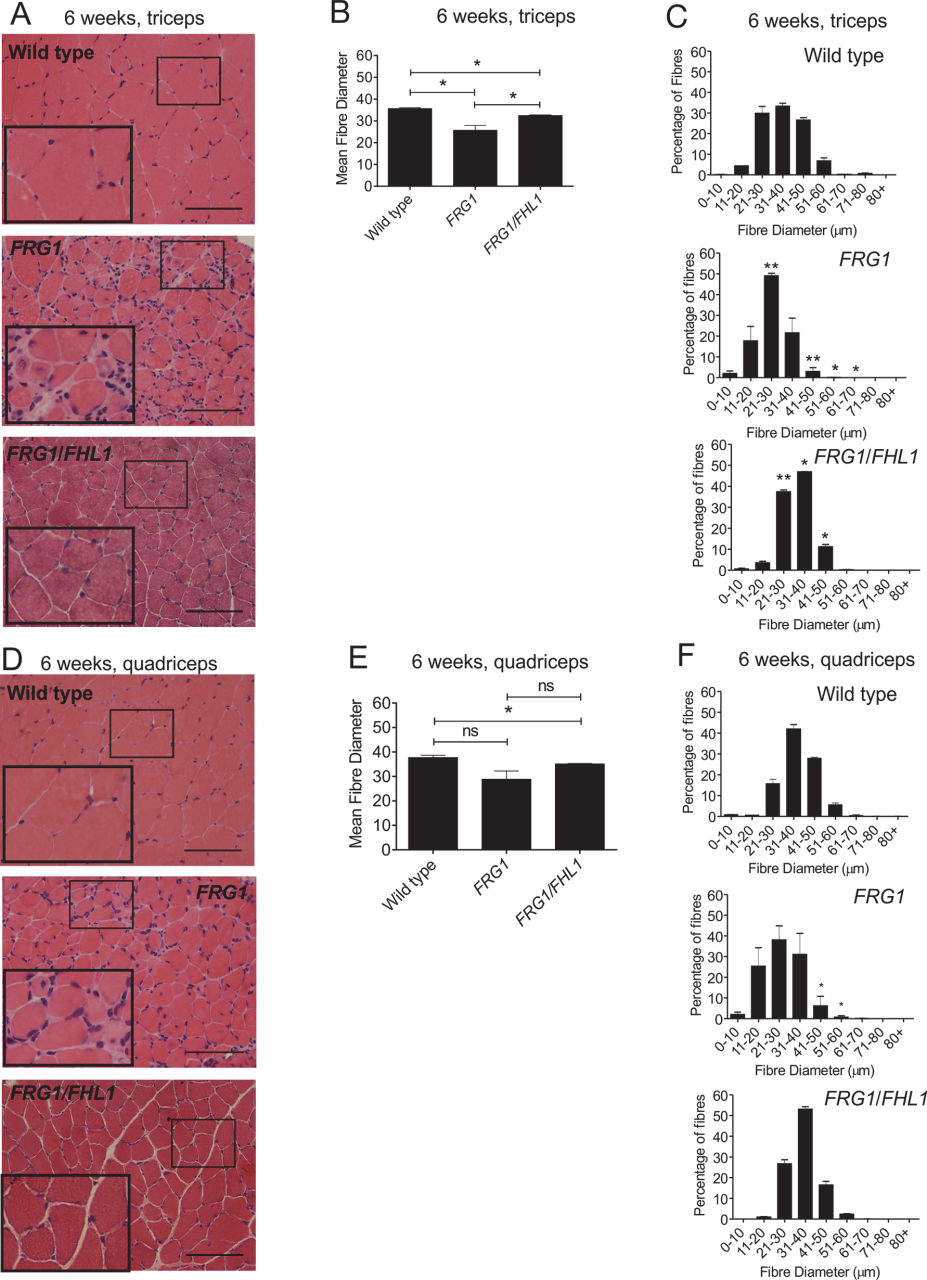
FHL1 improves muscle pathology in dystrophic *FRG1* mice. Representative images of transverse muscle sections from the triceps (A) or quadriceps (D) muscles of 6-week-old wild type, *FRG1* and *FRG1*/*FHL1* mice stained with H&E. Boxed region indicates area shown in high magnification image inset. Mean myofiber diameter from the triceps (B) and quadriceps (E) was measured for wild type, *FRG1* and *FRG1*/*FHL1* mice. Histograms showing the frequency of individual muscle fiber diameters from the triceps (C) or quadriceps (F) of wild type, *FRG1* and *FRG1*/*FHL1* mice. 500–1000 muscle fibers were measured per muscle for each mouse; Wild type (n = 3 mice), *FRG1* (n = 4 mice) and *FRG1*/*FHL1* (n = 4 mice). Data represent mean ± SEM; *p<0.05; **p<0.005 determined by two-tailed Student’s T-test. In (C) and (F), asterisks in *FRG1* histograms indicate significant differences between *FRG1* and wild type mice; Asterisks in *FRG1*/*FHL1* histogram indicate significant differences between *FRG1*/*FHL1* and *FRG1* mice. Scale bars = 100μm.

The dystrophic features reported in muscle from *FRG1*-transgenic mice include variation in muscle fiber size, centralized myonuclei and the presence of fibrosis [2] and were observed in H & E stained transverse muscle sections; (Fig. 4A and 4D, middle panels). We next examined specific features of the dystrophic phenotype by examining the quadriceps and triceps muscles, tissues representing high and intermediate degrees of muscle disease in the *FRG1* mouse respectively [2]. These muscles, particularly the quadriceps, have been used in several previous studies to characterize the dystrophic phenotype of the *FRG1* mouse including the examination of satellite cell and myoblast function [33]. Significantly, FHL1 expression in the triceps and quadriceps of 6-week-old *FRG1* mice resulted in significantly improved pathology, with uniform tightly packed fibers and reduced fibrotic tissue (Fig. 4A and 4D, lower panels). Mean myofiber diameter was decreased in the *FRG1* triceps and quadriceps compared to wild type (Fig. 4B and 4E) consistent with the presence of myofiber atrophy reported in this dystrophic model [2]. Myofiber atrophy was further examined by assessing the frequency of individual fiber diameters. In wild type mouse triceps and quadriceps muscles, fibers were 21–50μm in diameter, with some as large as 60–80 μm (Fig. 4C and 4F). *FRG1* muscle showed a significant shift in myofiber size towards an increase in smaller atrophic fibers (11–30 μm) and a corresponding reduction in larger fibers > 30 μm (Fig. 4C and 4F). Importantly, FHL1 expression in *FRG1* mouse triceps muscle resulted in a significant increase in both mean myofiber diameter (Fig. 4B) and also the frequency of larger myofibers > 30 μm (Fig 4C). *FRG1*/*FHL1* muscle also exhibited a 3–4-fold reduction in the proportion of small atrophic fibers (>20 μm) (Fig. 4C). In 12-week-old adult *FRG1*/*FHL1* mice the increase in myofiber size induced by FHL1 expression was sustained, although this was not as substantial as at 6 weeks (S1 Fig.). A previous study has examined the progressive course of muscle disease in the *FRG1*-transgenic mouse model between the ages of 3–14 weeks [33]. The dystrophic phenotype in the *FRG1*-transgenic mouse does not become apparent until 6-weeks of age and progressively worsens thereafter. Therefore, the improvement in mean fiber diameter observed at 6 weeks in the *FRG1/FHL1* mice may not be as prominent at 12 weeks due to the progressive worsening of the dystrophic FRG1 phenotype, such that in older mice only a partial rescue was observed. Collectively these results show that FHL1 promotes increased myofiber size most significantly during the growth phase of juvenile *FRG1* mice.

### FHL1 reduces fibrosis in *FRG1* mice

**Fig 5:**
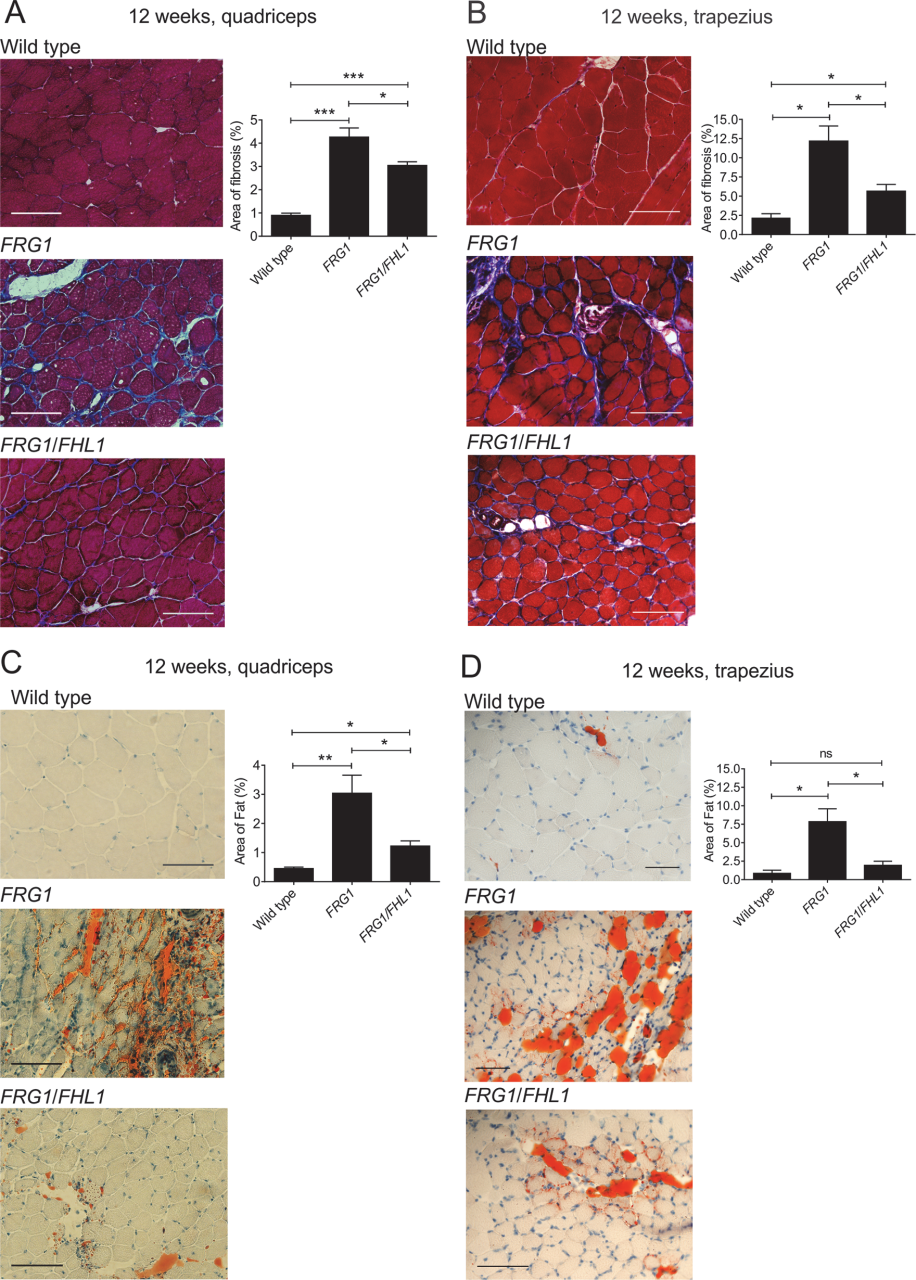
FHL1 reduces fibrosis and fat deposition in dystrophic *FRG1* mice. Representative images of transverse sections of (A) quadriceps and (B) trapezius muscle from 12-week-old wild type, *FRG1* and *FRG1*/*FHL1* mice stained with Masson’s trichrome to detect fibrosis within muscle. The percentage area of fibrosis staining in muscle was quantified from wild type (n = 4–6), *FRG1* (n = 4–6) and *FRG1*/*FHL1* (n = 5–6) mice. Representative images of transverse muscle sections from the (C) quadriceps and (D) trapezius muscle stained with Oil Red O to detect fat deposits within muscle. The percentage area of fat deposition in muscle was quantified in wild type (n = 3–4), *FRG1* (n = 4) and *FRG1*/*FHL1* (n = 4) mice. Data represent the mean ± SEM; *p<0.05; **p<0.005; ***p<0.0005 determined by two-tailed Student’s T-test. Scale bars = 100μm.

Dystrophic muscle is progressively replaced with fibro-fatty tissue as a consequence of the diminished capacity of satellite cells to repair damaged muscle. This may be caused by several factors including, replicative aging leading to satellite cell senescence, an unfavorable microenvironment for satellite cell mediated muscle repair, or due to the failure of muscle precursor cells (myoblasts) to differentiate efficiently [65]. The accumulation of fibrosis and fat in muscle was examined in two of the most severely affected muscles in *FRG1*-transgenic mice, the quadriceps and trapezius, which exhibit the greatest extent of fibrosis [2] and are therefore best suited to determine if FHL1 ameliorates this important disease feature. Masson’s trichrome staining of 12-week-old *FRG1* muscle revealed extensive fibrosis between muscle fibers, occupying 4% of the total muscle area in quadriceps and 12.5% in the trapezius, and was largely absent in wild type muscle (Fig. 5A-B). Notably, expression of FHL1 in both quadriceps and trapezius *FRG1* muscle resulted in a significant decrease in fibrosis, relative to *FRG1* muscle in the absence of exogenous FHL1 (Fig. 5A-B). Oil Red O staining of fat revealed a ∼6-fold increase in fat deposition in *FRG1* muscles relative to wild type and this was reduced in *FRG1*/*FHL1* muscle (Fig. 5C-D). Therefore, FHL1 expression is sufficient to reduce the accumulation of fibro-fatty scar tissue, not only in the quadriceps of *FRG1*-transgenic mice, but also in the trapezius where fibrosis and fat deposition are prominent pathological features.

### Analysis of muscle function in *FRG1 versus FRG1/FHL1* mice

Analysis of several parameters was undertaken to compare muscle function in wild type, *FRG1* and *FRG1/FHL1* mice including, maximum force, specific (normalized) force, frequency force relationship and resistance to fatigue (n ≥ 5 mice per genotype). For all parameters measured, a marked decrease in muscle function in *FRG1* mice was observed compared to wild type, however no improvement was observed in *FRG1/FHL1* mice (S2 Fig.). This indicates that despite several lines of evidence showing FHL1 promotes an improvement in *FRG1* muscle pathology, this was not sufficient to improve muscle function.

### FHL1 does not alter satellite cell number or activation in *FRG1 mice*

Previous studies have shown *FRG1* mice exhibit decreased satellite cell proliferation and activation, which are thought to contribute to the dystrophic phenotype by restricting muscle growth and repair [33,66]. In contrast, we have shown muscle from *FHL1*-transgenic muscle exhibits increased numbers of satellite cells (in the absence of muscle damage) and features of enhanced myoblast fusion [48]. Therefore, we systematically examined the number of satellite cells, satellite cell activation, myoblast differentiation and fusion in *FRG1 versus FRG1/FHL1* muscle to determine the mechanism(s) by which FHL1 reduces disease severity. Mice were examined at 6- and 12-weeks of age, representing both the early mild and late advanced stages of the dystrophic phenotype respectively in the *FRG1* mice [33]. It should be noted that both the *FRG1*- [2] and *FHL1*-transgenic [48] mice were generated using the human skeletal actin (HSA)-promoter, which is active in differentiating myoblasts and mature muscle fibers, but not satellite cells [67,68]. Despite this, changes to the satellite cell population have been reported in both the *FRG1* [33,66] and *FHL1* [48] mouse models, via unknown mechanisms. Indeed it is widely accepted that extrinsic factors which are secreted from other sources including muscle fibers and resident non-muscle cells can influence satellite cell and myoblast function [69–71]. Therefore, a comparison of satellite cells and myoblasts in the *FRG1 versus FRG1/FHL1* mouse models is a valid and necessary approach to understand how FHL1 expression can rescue the *FRG1* dystrophic phenotype.

**Fig 6:**
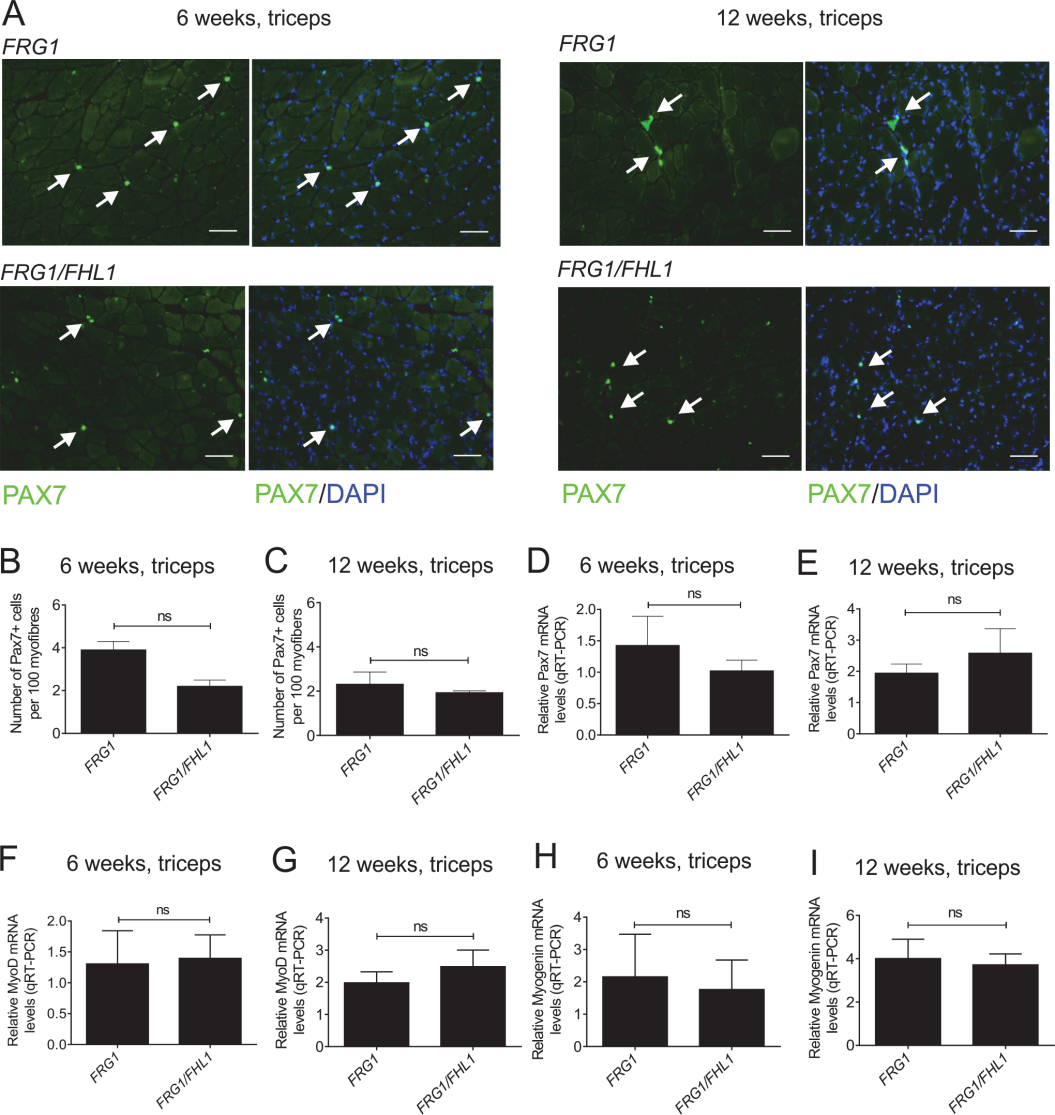
FHL1 does not alter satellite cell number or markers of satellite cell activation (MyoD) or differentiation (myogenin) in the triceps of *FRG1* mice. (A) Transverse muscle sections from the triceps of wild type, *FRG1* and *FRG1/FHL1* mice (aged 6- and 12-weeks) were co-stained with a satellite cell specific marker (pax7) and DAPI to detect nuclei. Arrows indicate pax7+ satellite cells. Boxed region indicates area shown in high magnification image inset. Scale bars = 100μm. The number of pax7+ satellite cells per 100 myofibers was counted for the triceps in mice aged (B) 6-weeks (*FRG1* n = 3 and *FRG1/FHL1* n = 3) and (C) 12-weeks (*FRG1* n = 4 and *FRG1/FHL1* n = 4). Quantitative RT-PCR analysis of pax7 (D- 6 weeks, E- 12 weeks) MyoD (F- 6 weeks, G- 12 weeks) and myogenin (H- 6 weeks, I- 12 weeks) mRNA in wild type, *FRG1* and *FRG1/FHL1* (n = 7 mice/genotype) triceps muscle. Data represent the mean ± SEM; ns not significant; *p<0.05; **p<0.001 determined by two-tailed Student’s T-test.

Satellite cell number was examined by immunostaining transverse muscle sections for the marker, Pax7 (paired-box gene 7) [72] and at both 6- and 12-weeks of age, no difference was observed between *FRG1 versus FRG1/FHL1* muscle (Fig. 6 A-C triceps; S3 A–C Fig. quadriceps). This result was confirmed by qRT-PCR analysis of Pax7 mRNA (Fig. 6 D-E triceps; S3 D–E Fig. quadriceps). Satellite cell activation was examined by qRT-PCR analysis of MyoD mRNA [73], revealing no significant differences between *FRG1* and *FRG1/FHL1* muscle (Fig. 6 F-G triceps; Supplementary S3 F–G Fig., quadriceps). This was confirmed by Pax7/MyoD double immunofluorescent labeling of muscle sections, which revealed no difference in the Pax7^+^/MyoD^+^ or Pax7^-^/MyoD^+^ populations in *FRG1 versus FRG1/FHL1* muscle (S4 Fig.; triceps muscle shown). Expression of myogenin, which marks the initiation of myoblast differentiation [48], was also unchanged between *FRG1* and *FRG1/FHL1* muscle (Fig. 6 H-I triceps; S3 H–I Fig. quadriceps). Collectively, this suggests that FHL1 does not enhance the activation of satellite cells or myoblast differentiation above that observed in dystrophic *FRG1* muscle.

### FHL1 increases myoblast fusion in *FRG1* mice

The terminal phase of myoblast differentiation involves cell fusion to form multi-nucleated myotubes. A previous study has established that *FRG1* mice have a myoblast fusion defect in skeletal muscle *in vivo* [33]. This was shown by examining young *FRG1* mice aged 4 weeks, just prior to development of the dystrophic phenotype at 6 weeks, which exhibit a reduction in the number of nuclei per muscle fiber, compared to age-matched wild type mice. This indicates that in young pre-dystrophic *FRG1* muscle, fewer myonuclei are incorporated into growing muscle fibers via myoblast fusion, causing a reduction in postnatal muscle growth.

After 6 weeks of age, the dystrophic phenotype develops in *FRG1* mice and hence there is a requirement for muscle regeneration, which does not occur in the healthy muscle from wild type mice. Therefore after 6 weeks of age *FRG1* mice exhibit an increase in the proportion of muscle fibers with centralized nuclei compared to wild type, in the latter there is no muscle disease and hence no requirement for muscle regeneration or increased myoblast fusion.

**Fig 7:**
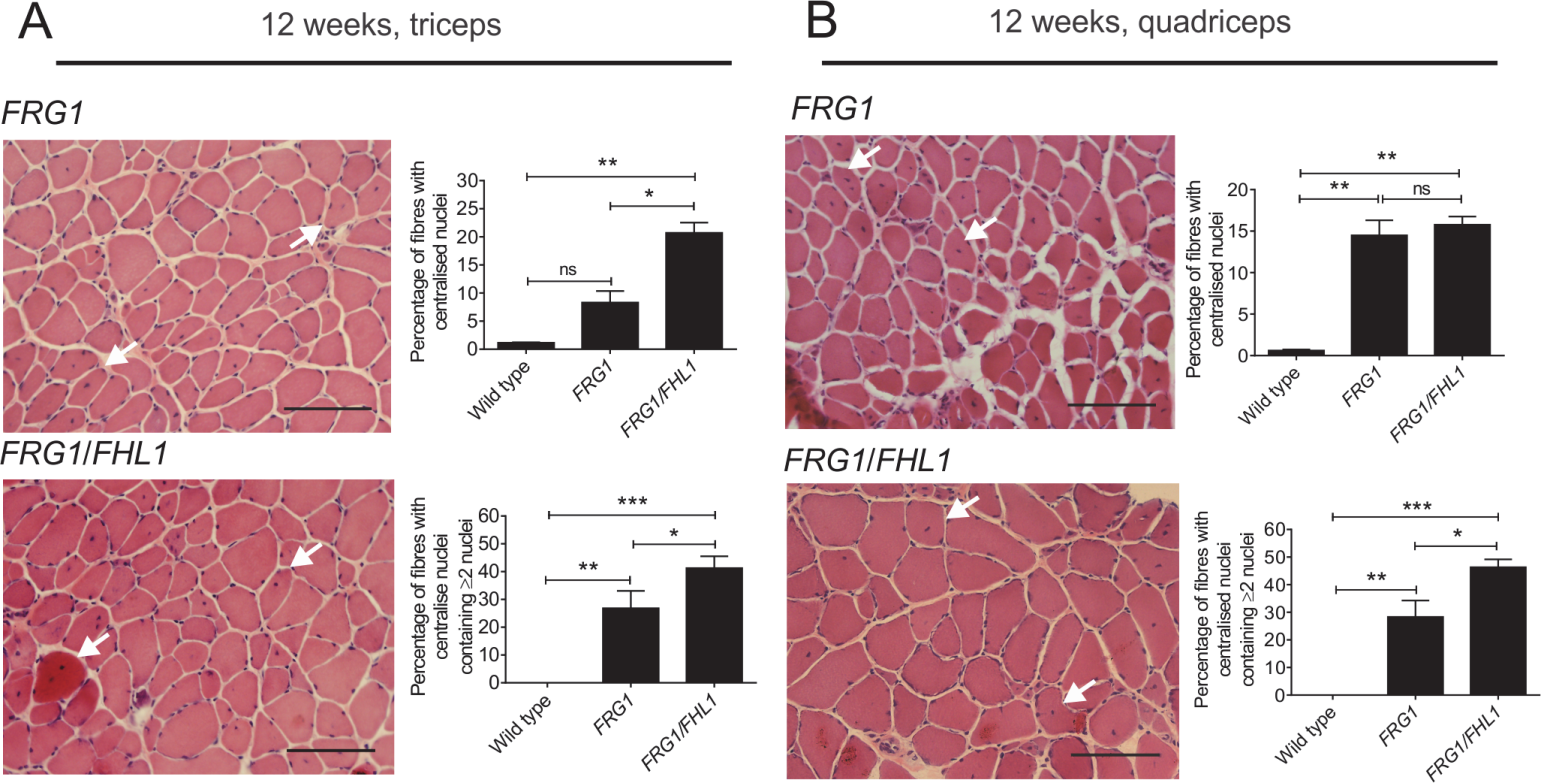
FHL1 increases the proportion of muscle fibers with centralized nuclei in *FRG1* mice. Representative images of transverse sections of triceps (A) or quadriceps (B) muscles from 12-week-old *FRG1* and *FRG1/FHL1* mice stained with H&E. Arrows indicate myofibers with centralized nuclei, an indicator of myoblast fusion *in vivo*. Scale bars = 100μm. For both triceps and quadriceps muscles the percentage of muscle fibers with centralized nuclei was quantified for wild type (n = 3), *FRG1* (n = 4) and *FRG1*/*FHL1* (n = 4) mice. The subset of these fibers containing multiple centralized nuclei was further quantified at 12 weeks of age. Data represent the mean ± SEM; ns not significant, *p<0.05; **p<0.005; ***p<0.0005 determined by two-tailed Student’s T-test.

**Fig 8:**
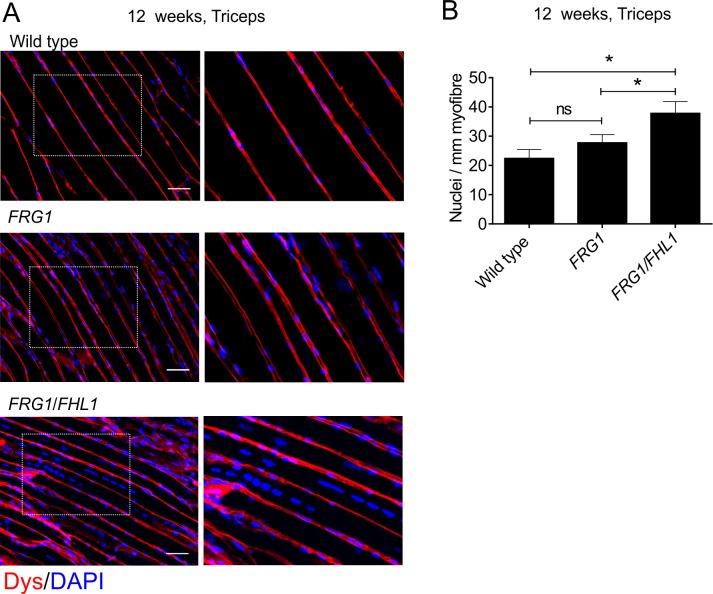
FHL1 enhances myoblast fusion in *FRG1* mice. (A) Representative images of longitudinal sections of triceps muscle from 12-week old wild type, *FRG1* and *FRG1/FHL1* mice co-stained for dystrophin to outline the muscle fiber membrane and DAPI to detect nuclei. Boxed region indicates area shown in high magnification image inset. Scale bars = 100μm. (B) The number of nuclei per mm of muscle fiber was counted as a measure of myoblast fusion at 12 weeks (wild type n = 3, *FRG1* n = 4, *FRG1/FHL1* n = 4). Data represent the mean ± SEM; ns not significant, *p<0.05determined by two-tailed Student’s T-test.

In previous work, we have shown that FHL1 enhances myoblast fusion *in vitro* [48]. Therefore, we examined if expression of FHL1 in *FRG1/FHL1* mice was sufficient to enhance the level of myoblast fusion above that observed in *FRG1* mice. Myoblast fusion was assessed in muscle *in vivo* by examining standard markers of this process; quantifying the proportion of fibers with centrally located nuclei and also the average number of myonuclei per muscle fiber [74]. First, we determined the proportion of total fibers containing centralized nuclei by analysis of H&E stained transverse sections of triceps and quadriceps muscles from adult 12-week-old mice. This analysis revealed *FRG1*/*FHL1* muscles showed a significant increase in the proportion of myofibers with centralized nuclei relative to *FRG1* mice in the triceps, but not quadriceps muscle (Fig. 7A-B). Next, we determined what subset of these fibers with centralized nuclei contained multiple centralized nuclei, as a further indicator of enhanced myoblast fusion [70,75]. This was increased in the *FRG1/FHL1* triceps and quadriceps muscles compared to *FRG1* muscle (Fig. 7A-B), further supporting the contention that FHL1 promotes increased nuclei addition to muscle fibers via enhanced myoblast fusion. We next quantified the number of nuclei per mm length of myofiber using longitudinal sections of the triceps, a dystrophic muscle in *FRG1* mice [2]. The triceps long-head represents a single muscle group with all fibers running in parallel, allowing for accurate quantification of the number of myonuclei along the entire length of individual fibers.Analysis of longitudinal triceps muscle sections revealed the number of nuclei per mm of muscle fiber length was significantly increased in 12-week-old *FRG1/FHL1* mice relative to both *FRG1* and wild type mice (Fig. 8). Therefore, FHL1 enhances myoblast fusion during disease progression in *FRG1* muscle.

### FHL1 does not alter expression of the methyltransferase Suv4–20h1 or differentiation inhibitor Eid3 in *FRG1* mice

It was recently reported that the histone methyltransferase Suv4–20h1 is a gene-specific repressor required for myogenic differentiation [35]. FRG1 over-expression interferes with the repressive action of Suv4–20h1 leading to aberrant upregulation of the inhibitor, EP300 interacting inhibitor of differentiation 3 (Eid3), resulting in myoblast differentiation defects [35]. To determine if FHL1 enhances myoblast fusion through altering expression of Suv-20h1 or Eid3, we performed qRT-PCR analysis on triceps and quadriceps muscle from 6- and 12-week-old mice (S5 Fig.). We found no differences in Suv4–20h1 mRNA levels between wild type, *FRG1* or *FRG1/FHL1* mice, but observed a significant increase in Eid3 mRNA levels and protein expression in *FRG1* muscle relative to wild type muscle (S4 E–I Fig.), as previously reported in *FRG1* mice and FSHD muscle [35]. However, the levels of Eid3 remained elevated in *FRG1/FHL1* muscle. Therefore FHL1 does not enhance myoblast fusion through alteration of the FRG1/Suv4–20h1/Eid3 pathway.

### *FRG1* myoblasts exhibit a myoblast fusion defect that is rescued in *FRG1*/*FHL1* myoblasts

**Fig 9:**
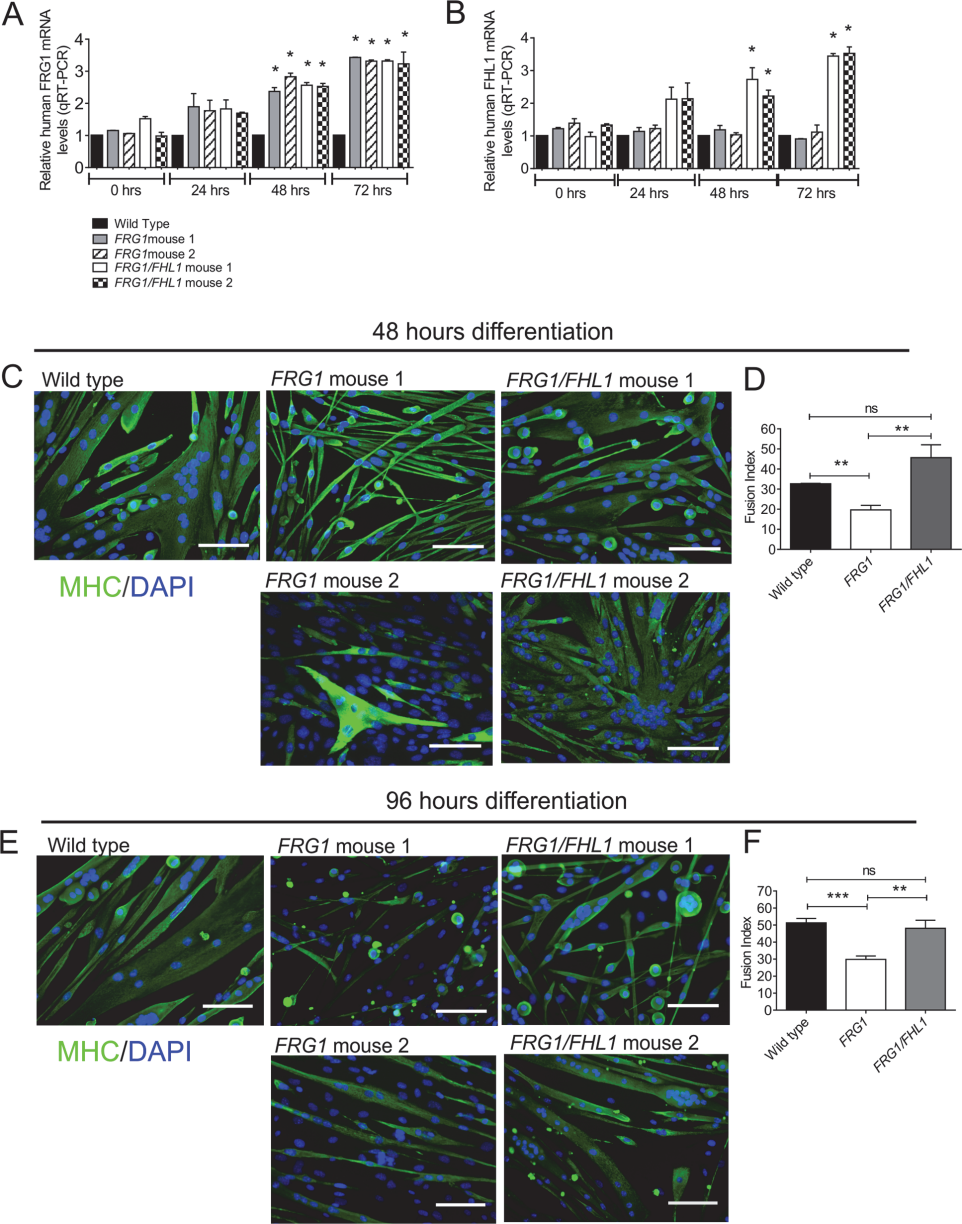
*FRG1* myoblasts exhibit a fusion defect that is rescued in *FRG1*/FHL1 myoblasts. Quantitative RT-PCR analysis of human FRG1 (A) and human FHL1 (B) mRNA in primary mouse myoblasts isolated from wild type mice, and two *FRG1* (1 and 2) and two *FRG1/FHL1* (1 and 2) mice. Data represent the mean +/- SEM of n = 3 independent experiments and was standardized to GAPDH and expressed relative to control wild type myoblasts. *p<0.05. Representative images of primary myoblasts from wild type, *FRG1* and *FRG1*/*FHL1* mice following 48 hours (C) or 96 hours (E) differentiation. Cultures were stained with the differentiation marker MHC (green) and DAPI for detection of the fusion index; the percentage of total nuclei localized within MHC-positive myotubes after 48 hours (D) and 96 hours (F) differentiation. Data represent the mean ± SEM from n = 3 independent experiments; wild type (n = 1); *FRG1* (n = 2); *FRG1*/*FHL1* (n = 2); ns not significant, **p<0.005, ***p<0.0001 determined by two-tailed Students T-test. Scale bars = 100μm.

Primary myoblasts isolated from neonatal mice are routinely used for comparison of differentiation and fusion potential in healthy and disease models [76–78]. This approach has also been recently reported inFSHD disease mouse model, the *DUX4* transgenic mouse [22]. Here, we compared the fusion of primary myoblasts isolated from wild type, *FRG1* and *FRG1/FHL1* mice. FRG1 and FHL1 are under control of the human skeletal muscle actin (HSA) promoter, which should only be active in mature muscle fibers or myoblasts post induction of differentiation [79,80]. In our study, qRT-PCR analysis using human-specific primers (to distinguish from endogenous murine transcripts) confirmed that both transgene-derived FRG1 and FHL1 mRNA were not induced in myoblast cultures until 48 hours differentiation consistent with the reported activation of the HSA promoter at this time point. We observed a 2–3-fold increase in FRG1 mRNA in all differentiating myoblast cultures from *FRG1* and *FRG1/FHL1* mice (Fig. 9A), and a 2–4-fold increase in FHL1 mRNA in differentiating myoblasts from *FRG1/FHL1* mice (Fig. 9B).

Here we established that the dystrophic pathology of *FRG1* mice is rescued by FHL1 through enhancement of myoblast fusion. In a proof of principle experiment the differentiation of primary myoblasts isolated from *FRG1 versus FRG1*/*FHL1* mice were compared. Myoblasts isolated from wild type mice fused to form long multinucleated MHC-positive myotubes, but many *FRG1* myoblasts remained as mononucleated cells that did not fuse, however, occasional thin MHC-positive myotubes containing small numbers of nuclei formed (Fig. 9C and 9E). This defect in myoblast fusion was rescued in *FRG1*/*FHL1* myoblasts, which formed long multinucleated MHC-positive myotubes (Fig. 9C and 9E) with a 2 fold-increase in the fusion index relative to *FRG1* mouse-derived myoblasts (Fig. 9D and 9F), both at 48 hours and 96 hours differentiation. Therefore, myoblasts isolated from *FRG1*/*FHL1* mice fused more efficiently than those from dystrophic *FRG1* mice

## Discussion

Here we investigated the selective involvement of FRG1 in regulating myoblast differentiation, revealing a significant myoblast fusion defect in C2C12 myoblasts overexpressing FRG1, and in primary myoblasts derived from *FRG1*-transgenic-mice. As such, we predicted in proof of principle experiments that agents which promote myoblast fusion may rescue the *FRG1*-transgenic muscle phenotype. Importantly, our study reveals that FHL1, a positive regulator of muscle hypertrophy and myoblast fusion, reduces muscle wasting and improves the muscular dystrophy phenotype observed in *FRG1* mice by rescuing the FRG1-induced myoblast fusion defect. Critically, FHL1 is able to circumvent the myoblast fusion defects in *FRG1* mice, despite not directly correcting an underlying molecular cause, the inhibitory FRG1/Suv4–20h1/Eid3 pathway.

### FRG1 overexpression causes a myoblast fusion defect

Myoblast fusion is essential for skeletal muscle formation during development, post-natal muscle growth and for the regeneration of skeletal muscle. Increasing evidence supports the contention that defects in satellite cells and myoblasts contribute to disease pathogenesis in βconflicting and can vary with age and disease severity, and also differences have been noted between *in vivo* and *in vitro* analyses of satellite cell proliferation [33,66]. Satellite cell activation is also impaired in *FRG1* muscle leading to impaired muscle growth and regeneration that precedes the development of the dystrophic phenotype [33]. Increased expression of FRG1 impairs the terminal differentiation of myoblasts [33] through interaction with the histone methytransferase Suv4–20h1 and de-repression of Eid3 [35]. In these recent reports, the differentiation defect of myoblasts overexpressing FRG1 was assessed by examining terminally differentiated myoblasts expressing the marker MHC and also by quantifying the nuclear fusion index (number of nuclei/MHC+ myotube). Our study has undertaken a more extensive characterization of myoblast differentiation in C2C12 myoblasts overexpressing FRG1 and as such has expanded upon the current knowledge of the effect of increased FRG1. This new data demonstrates that FRG1 overexpression does not impair the initiation of myoblast differentiation, as there was no decrease in the expression of the master regulator, myogenin [81]. However in myoblasts over-expressing FRG1, defects in the latter stages of differentiation, and a significant impairment of myoblast fusion was observed. This myoblast fusion defect was shown in C2C12 myoblasts overexpressing FRG1 and also primary myoblasts derived from *FRG1*-transgenic mice. Therefore our data, together with recent literature, firmly supports a model whereby defects in myoblast function underlie the pathogenesis of muscle disease in the *FRG1*-transgenic mouse. In our study we rigorously tested this hypothesis by attempting to rescue the dystrophic phenotype in *FRG1* mice by enhancing muscle fusion via FHL1 expression.

### FHL1 rescues the *FRG1* muscular dystrophy phenotype

If myoblast fusion defects do play an important role in the pathogenesis of muscle disease, then it is reasonable to consider that circumventing this defect has potential as a therapeutic strategy. Ours is the first study to test the hypothesis that increasing myoblast fusion circumvents the dystrophic phenotype using the *FRG1* mouse as a model. This was achieved by crossing the *FRG1*-transgenic mouse with a mouse model expressing increased levels of FHL1, a known activator of myoblast fusion [48]. *FHL1*-transgenic mice develop muscle hypertrophy, oassociated with increased muscle stem cells (satellite cells), increased muscle strength and protection from age-related muscle weakness [48]. Over-expression of FHL1 promotes secondary myoblast fusion, that is, the fusion of myoblasts with nascent myotubes [48]. FHL1 binds and co-activates the transcription factor NFATc1 (nuclear factor of activated T-cells) [48]. Activation of the calcineurin/NFAT pathway is essential for embryonic skeletal muscle development, hypertrophy, regeneration and protection against muscle atrophy [48,82–84]. Central to the role calcineurin/NFAT plays in regulating many of these processes is the activation of myoblast fusion by this pathway [48,85,86].

Notably, we demonstrated that *FRG1* mice overexpressing FHL1 show significant improvement in the dystrophic phenotype, with reduced fibrosis and fat deposition associated with increased muscle mass and myofiber size relative to *FRG1* mice. FHL1 did not alter satellite cell number, satellite cell activation or myogenic differentiation and therefore this suggests that FHL1 does not ameliorate the dystrophic phenotype in *FRG1* mice through recovery of satellite cell function. However, we did observe an increase in the proportion of muscle fibers with one or more centralized nuclei and an increase in the number of nuclei along the length of individual muscle fiber in *FRG1/FHL1* mice relative to *FRG1*, and both parameters are accepted indicators of enhanced myoblast fusion *in vivo* [74,87]. Critically, in a key proof of principle experiment, myoblasts isolated from *FRG1*/*FHL1* mice exhibited rescue of the myoblast fusion defect that was observed in *FRG1* mouse myoblasts. Therefore FHL1 ameliorates the dystrophic phenotype of *FRG1* mice by enhancing myoblast fusion and thereby maintenance of muscle mass.

### FHL1 does not improve muscle function in *FRG1* mice

FHL1 promoted an improvement to the phenotype and muscle pathology in *FRG1* mice via enhanced myoblast fusion. However despite this, FHL1 expression was not sufficient to recover muscle function in *FRG1* mice. In human and mouse muscle FRG1 localizes to the contractile sarcomere [88] and one possible explanation for the lack of functional improvement in *FRG1/FHL1* muscles is the recently reported role for FRG1 in regulating muscle contractility. *FRG1*-transgenic mice express an abnormal troponin T isoform in fast skeletal muscles due to aberrant splicing of the *Tnnt3* mRNA [89]. As a result muscles from *FRG1*-transgenic mice exhibit a reduction in muscle strength and contractile properties due to an altered MyHC/actin ratio and reduced sensitivity to Ca^2+^. This decreased sensitivity of fast muscle fibers to Ca^2+^ is caused by expression of the abnormal troponin. FRG1 also binds actin and has a role in stabilizing actin filaments [5,6]. These results suggest that overexpression of FRG1 has two consequences in muscle, defects in myoblast fusion and impaired muscle contractility. In the current study we provide evidence that expression of FHL1 is sufficient to correct the myoblast fusion defect caused by FRG1 overexpression in *FRG1*-transgenic mice. However it is possible that an underlying defect in contractile proteins still exists in *FRG1/FHL1* mice, which precludes functional improvement.

### FHL1 alleviates myoblast fusion defects

There is an overt lack of effective treatments for many muscle diseases, including FSHD. The identification of myoblast differentiation and fusion defects in FSHD and also in an increasing number of other muscle diseases including Limb-girdle Muscular Dystrophy type 2B and Myotonic Dystrophy type 1 (DM1) [40,42,90], suggests that enhancing myoblast fusion may form part of a potential therapeutic strategy. There is a strong association between FHL1 expression/function and muscle health. The identification of multiple *FHL1* mutations as causative for human muscle disease highlights the critical importance of FHL1 function for normal muscle homeostasis [44–47]. Myoblast fusion is regulated by the balance of FHL1 expression; increased FHL1 expression enhances myoblast fusion [48], whereas decreased FHL1 expression impairs myoblast [47,91]. The current study is the first to exploit this important regulatory function for FHL1 in controlling myoblast fusion in a muscular dystrophy model, revealing partial amelioration of the FSHD disease phenotype.

## Supporting Information

S1 Fig Analysis of muscle pathology in *FRG1/FHL1* mice at 12 weeks of age. Representative images of transverse muscle sections from the triceps (A) or quadriceps (D) muscles of 12-week-old wild type, *FRG1* and *FRG1*/*FHL1* mice stained with H&E. Boxed region indicates area shown in high magnification image inset. Mean myofiber diameter from the triceps (B) and quadriceps (E) was measured for wild type, *FRG1* and *FRG1*/*FHL1* mice. Histograms showing frequency of individual muscle fiber diameters from the triceps (C) or quadriceps (F) muscles of wild type, *FRG1* and *FRG1*/*FHL1* mice. 500–1000 muscle fibers were measured for per muscle for each mouse; Wild type (n = 3–4 mice), *FRG1* (n = 4 mice) and *FRG1*/*FHL1* (n = 5 mice) Data represent the mean ± SEM; *p<0.05; **p<0.005; ***p<0.0005 determined by two-tailed Student’s T-test. In (C) and (F), asterisks in *FRG1* histograms indicate significant differences between *FRG1* and wild type mice; Asterisks in *FRG1*/*FHL1* histogram indicate significant differences between *FRG1*/*FHL1* and *FRG1* mice. Scale bars = 100μm.
(TIF)

S2 Fig Measurement of muscle contractile parameters and fatigue resistance in wild type, *FRG1* and *FRG1/FHL1* mice. Maximum force (A), specific (normalized) force (B), frequency force relationship (C), and resistance to fatigue expressed both as a percentage of initial force (D), and as raw force (E) of TA muscles from 8-week old wild type, *FRG1* and *FRG1/FHL1* mice, measured *in situ*. Data represent the mean ± SEM from n ≥ 5 mice per genotype; ns not significant; *p<0.05; ***p<0.0005 determined by two-tailed Student’s T-test.
(TIF)

S3 Fig FHL1 does not alter satellite cell number or markers of satellite cell activation (MyoD) or differentiation (myogenin) in the quadriceps of *FRG1* mice. (A) Transverse muscle sections from the quadriceps of *FRG1* and *FRG1/FHL1* mice (aged 6- and 12-weeks) co-stained with a satellite cell specific marker (pax7) and DAPI to detect nuclei. Arrows indicate pax7+ satellite cells. Boxed region indicates area shown in high magnification image inset. Scale bars = 100μm. The number of pax7+ satellite cells per 100 myofibers was counted for the quadriceps in mice aged (B) 6 weeks (*FRG1* n = 3 and *FRG1/FHL1* n = 3–4) and (C) 12 weeks (n = 4/genotype). Quantitative RT-PCR analysis of pax7 (D- 6 weeks, E- 12 weeks) MyoD (F- 6 weeks, G-12 weeks) and myogenin (H- 6 weeks, I- 12 weeks) mRNA in wild type, *FRG1* and *FRG1/FHL1* (n = 7 mice/genotype) quadriceps muscle. Data represent the mean ± SEM; ns not significant; *p<0.05; **p<0.001 determined by two-tailed Student’s T-test.
(TIF)

S4 Fig FHL1 does not alter number of MyoD positive cells in the triceps or quadriceps of *FRG1* mice. Transverse muscle sections from the triceps of *FRG1* and *FRG1/FHL1* mice aged (A) 6- and (B) 12-weeks, co-stained with a marker for quiescent satellite cells (Pax7) and activated satellite cells (MyoD). Green arrows indicate Pax7^+^/MyoD^-^ cells; yellow arrows indicate Pax7^+^/MyoD^+^ cells; red arrows indicate Pax7^-^/MyoD^+^ cells. Boxed region indicates area shown in high magnification image at far right panel. Scale bars = 100μm. The number of Pax7^+^/MyoD^+^ cells per 100 myofibers from the triceps in mice aged (C) 6- and (D) 12-weeks; n = 3–4/genotype. The number of Pax7^-^/MyoD^+^ cells per 100 myofibers from the triceps muscle in mice aged (E) 6-and (F) 12-weeks; n = 3–4/genotype. Data represent the mean ± SEM and a Student’s T-test revealed no statistically significant difference (ns) between *FRG1* and *FRG1/FHL1* mice.
(TIF)

S5 Fig FHL1 does not alter expression of the methyltransferase Suv4–20h1 or differentiation inhibitor Eid3 in *FRG1* mice. Quantitative RT-PCR analysis of Suv4–20h1 mRNA in triceps muscle at 6-weeks (A) and 12-weeks (B) and in quadriceps muscle at 6-weeks (C) and 12-weeks (D) from wild type, *FRG1* and *FRG1/FHL1* mice (n = 7/genotype). Quantitative RT-PCR analysis of Eid3 mRNA in triceps muscle at 6-weeks (E) and 12-weeks (F) and in quadriceps muscle at 6-weeks (G) and 12-weeks (H) from wild type, *FRG1* and *FRG1/FHL1* mice (n = 7/genotype). Data represent the mean ± SEM; ns not significant; *p<0.05 determined by two-tailed Student’s T-test. (C) Western blot of Eid3 protein expression in wild type, *FRG1* and *FRG1/FHL1* triceps muscle. Immunoblotting for β-tubulin or staining of membranes with ponceau red was used as a protein loading control.
(TIF)

S1 Table List of antibodies used in this study. (DOC)

S2 Table Muscle weights from wild type, and *FRG1*- and *FRG1/FHL1*- transgenic mice at 6 weeks of age. (DOC)

S3 Table Muscle weights from wild type, and *FRG1*- and *FRG1/FHL1*- transgenic mice at 12 weeks of age. (DOC)

S1 Rawdata Western blot raw data. (PDF)
