# Supplementary figures and images for: FHL1 Reduces Dystrophy in Transgenic Mice Overexpressing FSHD Muscular Dystrophy Region Gene 1 (FRG1)
Source: PLoS One. 2015 Feb 19;10(2):e0117665. doi: 10.1371/journal.pone.0117665 (PMC4335040; doi:10.1371/journal.pone.0117665)

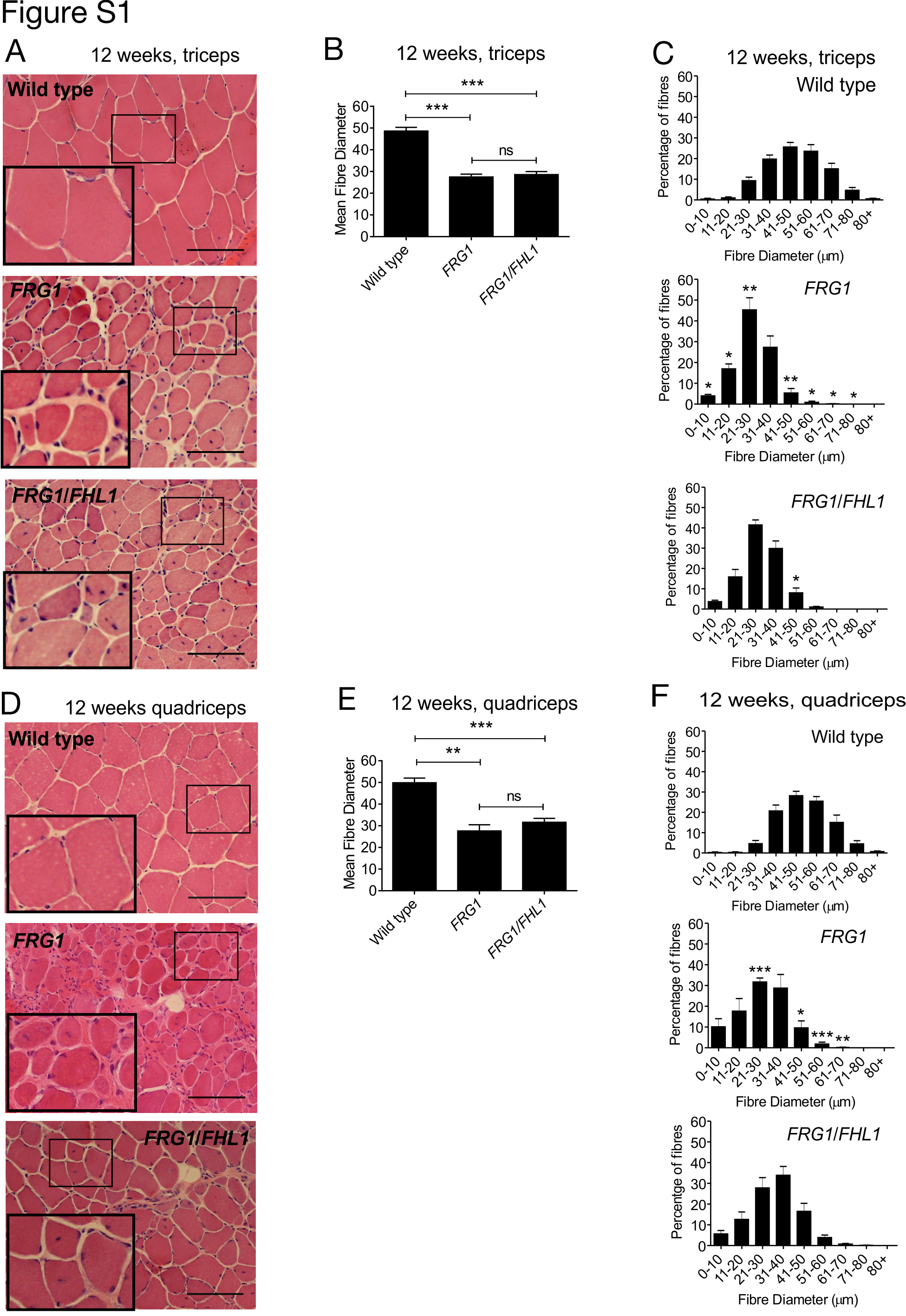

Supplement: S1 Fig — Representative images of transverse muscle sections from the triceps (A) or quadriceps (D) muscles of 12-week-old wild type, FRG1 and FRG1/FHL1 mice stained with H&E. Boxed region indicates area shown in high magnification image inset. Mean myofiber diameter from the triceps (B) and quadriceps (E) was measured for wild type, FRG1 and FRG1/FHL1 mice. Histograms showing frequency of individual muscle fiber diameters from the triceps (C) or quadriceps (F) muscles of wild type, FRG1 and FRG1/FHL1 mice. 500–1000 muscle fibers were measured for per muscle for each mouse; Wild type (n = 3–4 mice), FRG1 (n = 4 mice) and FRG1/FHL1 (n = 5 mice) Data represent the mean ± SEM; *p<0.05; **p<0.005; ***p<0.0005 determined by two-tailed Student’s T-test. In (C) and (F), asterisks in FRG1 histograms indicate significant differences between FRG1 and wild type mice; Asterisks in FRG1/FHL1 histogram indicate significant differences between FRG1/FHL1 and FRG1 mice. Scale bars = 100μm. (TIF) [file pone.0117665.s001.tif]

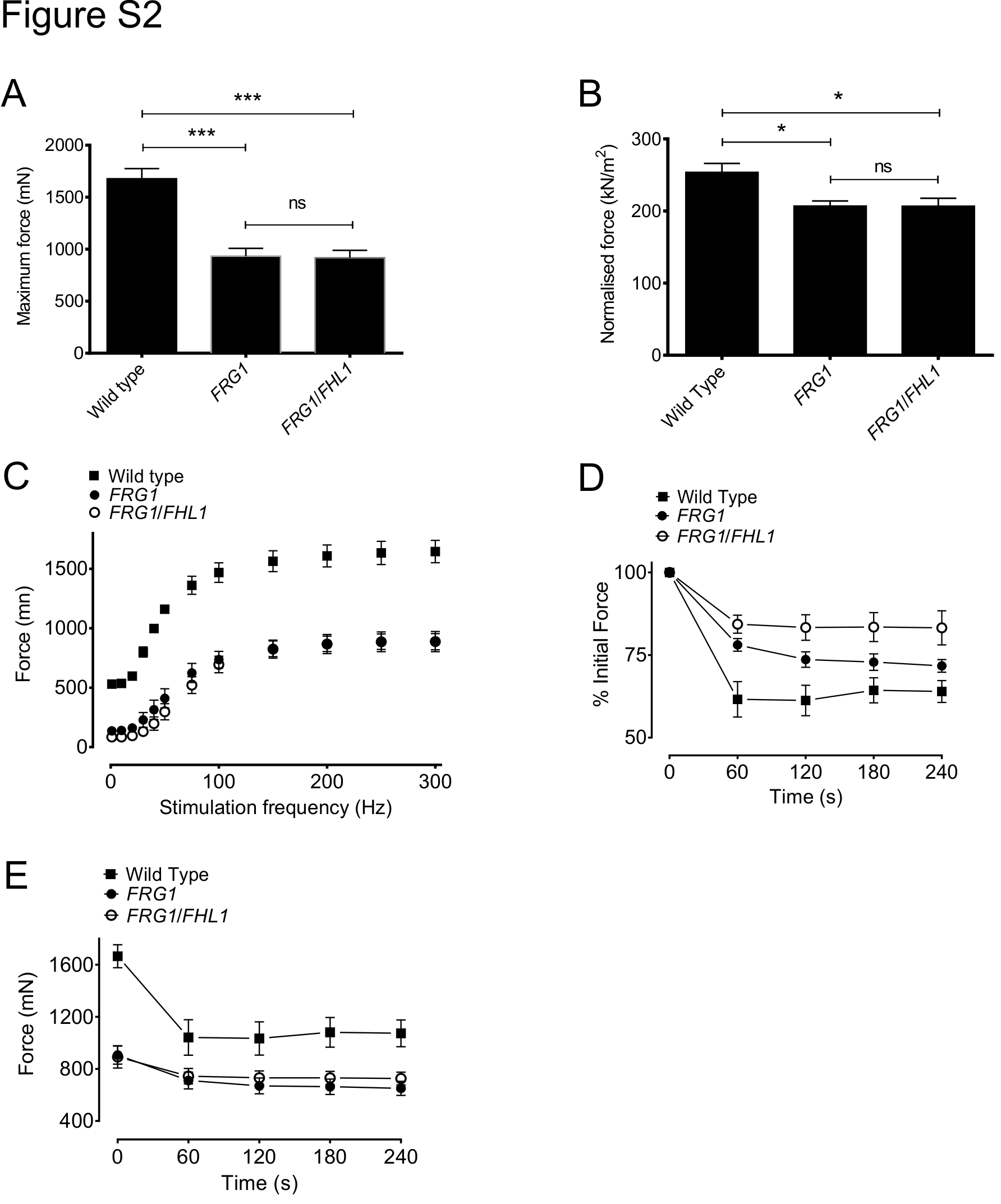

Supplement: S2 Fig — Maximum force (A), specific (normalized) force (B), frequency force relationship (C), and resistance to fatigue expressed both as a percentage of initial force (D), and as raw force (E) of TA muscles from 8-week old wild type, FRG1 and FRG1/FHL1 mice, measured in situ. Data represent the mean ± SEM from n ≥ 5 mice per genotype; ns not significant; *p<0.05; ***p<0.0005 determined by two-tailed Student’s T-test. (TIF) [file pone.0117665.s002.tif]

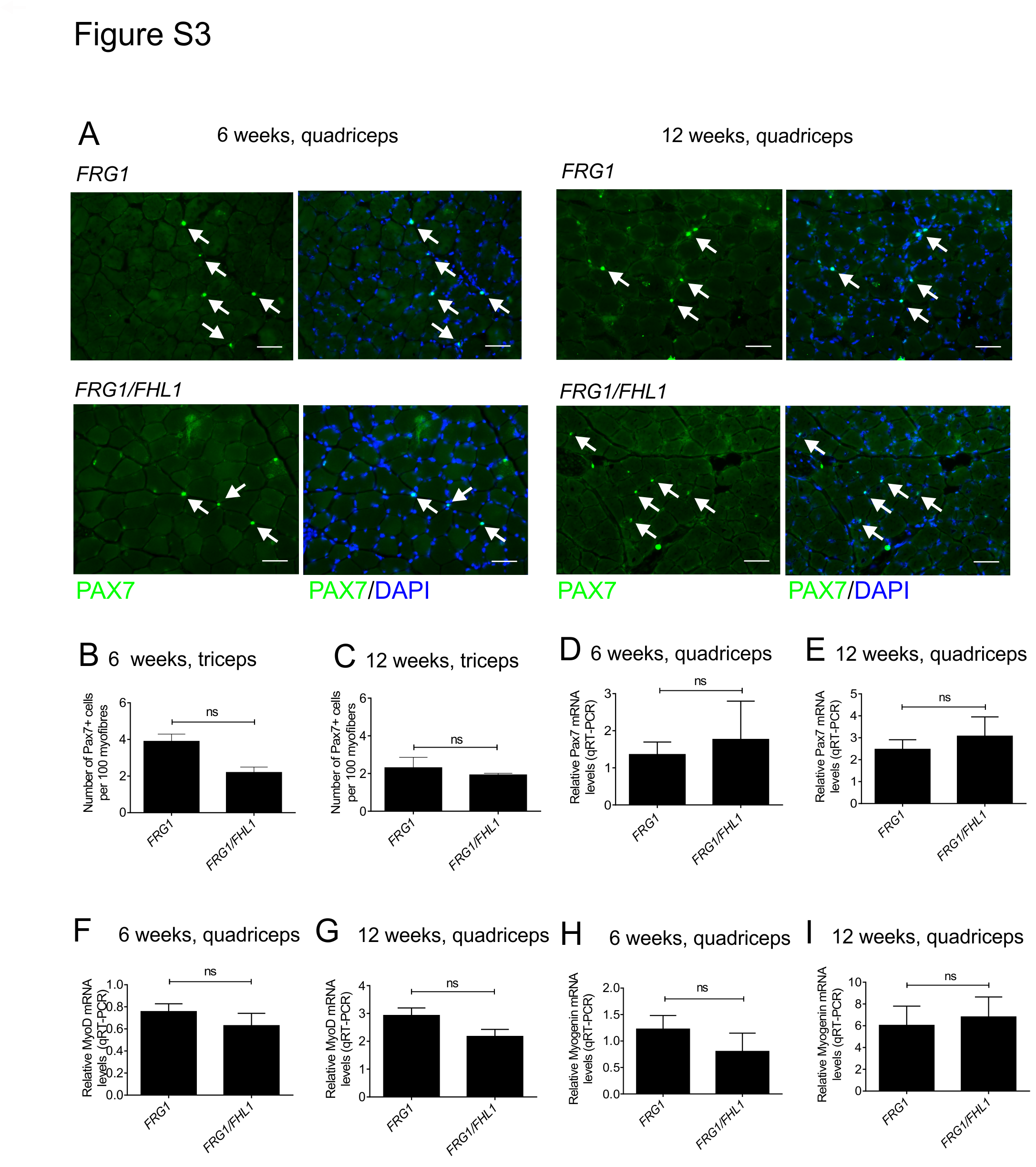

Supplement: S3 Fig — (A) Transverse muscle sections from the quadriceps of FRG1 and FRG1/FHL1 mice (aged 6- and 12-weeks) co-stained with a satellite cell specific marker (pax7) and DAPI to detect nuclei. Arrows indicate pax7+ satellite cells. Boxed region indicates area shown in high magnification image inset. Scale bars = 100μm. The number of pax7+ satellite cells per 100 myofibers was counted for the quadriceps in mice aged (B) 6 weeks (FRG1 n = 3 and FRG1/FHL1 n = 3–4) and (C) 12 weeks (n = 4/genotype). Quantitative RT-PCR analysis of pax7 (D- 6 weeks, E- 12 weeks) MyoD (F- 6 weeks, G-12 weeks) and myogenin (H- 6 weeks, I- 12 weeks) mRNA in wild type, FRG1 and FRG1/FHL1 (n = 7 mice/genotype) quadriceps muscle. Data represent the mean ± SEM; ns not significant; *p<0.05; **p<0.001 determined by two-tailed Student’s T-test. (TIF) [file pone.0117665.s003.tif]

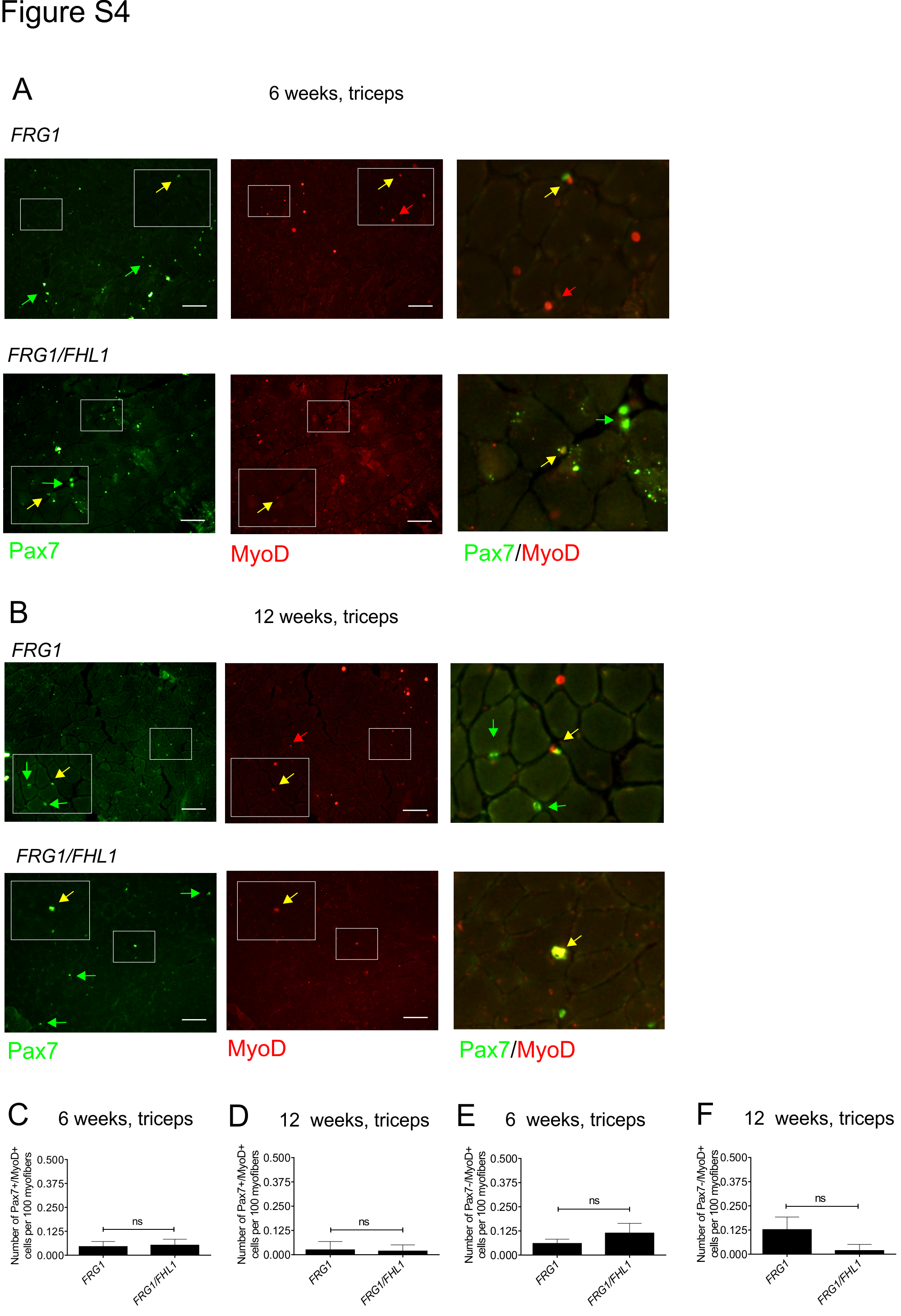

Supplement: S4 Fig — Transverse muscle sections from the triceps of FRG1 and FRG1/FHL1 mice aged (A) 6- and (B) 12-weeks, co-stained with a marker for quiescent satellite cells (Pax7) and activated satellite cells (MyoD). Green arrows indicate Pax7+/MyoD- cells; yellow arrows indicate Pax7+/MyoD+ cells; red arrows indicate Pax7-/MyoD+ cells. Boxed region indicates area shown in high magnification image at far right panel. Scale bars = 100μm. The number of Pax7+/MyoD+ cells per 100 myofibers from the triceps in mice aged (C) 6- and (D) 12-weeks; n = 3–4/genotype. The number of Pax7-/MyoD+ cells per 100 myofibers from the triceps muscle in mice aged (E) 6-and (F) 12-weeks; n = 3–4/genotype. Data represent the mean ± SEM and a Student’s T-test revealed no statistically significant difference (ns) between FRG1 and FRG1/FHL1 mice. (TIF) [file pone.0117665.s004.tif]

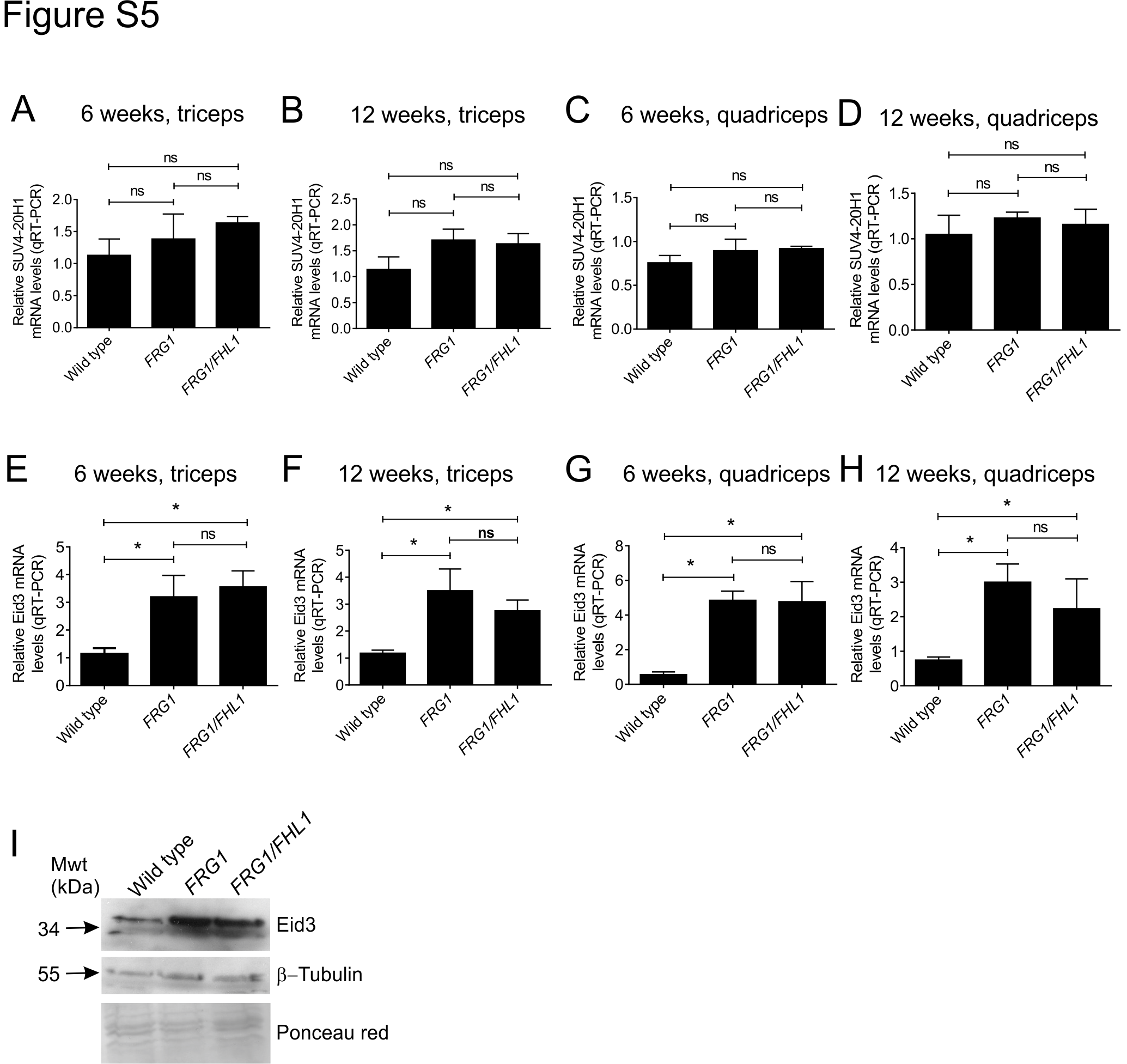

Supplement: S5 Fig — Quantitative RT-PCR analysis of Suv4–20h1 mRNA in triceps muscle at 6-weeks (A) and 12-weeks (B) and in quadriceps muscle at 6-weeks (C) and 12-weeks (D) from wild type, FRG1 and FRG1/FHL1 mice (n = 7/genotype). Quantitative RT-PCR analysis of Eid3 mRNA in triceps muscle at 6-weeks (E) and 12-weeks (F) and in quadriceps muscle at 6-weeks (G) and 12-weeks (H) from wild type, FRG1 and FRG1/FHL1 mice (n = 7/genotype). Data represent the mean ± SEM; ns not significant; *p<0.05 determined by two-tailed Student’s T-test. (C) Western blot of Eid3 protein expression in wild type, FRG1 and FRG1/FHL1 triceps muscle. Immunoblotting for β-tubulin or staining of membranes with ponceau red was used as a protein loading control. (TIF) [file pone.0117665.s005.tif]
