# Supplementary material for: FHL1 Reduces Dystrophy in Transgenic Mice Overexpressing FSHD Muscular Dystrophy Region Gene 1 (FRG1)
Source: PLoS One. 2015 Feb 19;10(2):e0117665. doi: 10.1371/journal.pone.0117665 (PMC4335040; doi:10.1371/journal.pone.0117665)
Supplement: S1 Table — (DOC) [file pone.0117665.s006.doc]

Table S1 Antibody Information

| **Antibody** | **Dilution** | **Supplier** | **Catalogue or clone number** | **Antigen used to raise antibody** | **Public Indentifier from Antibody Registry** | **Reference** |
| --- | --- | --- | --- | --- | --- | --- |
| Myogenin  Mouse monoclonal | 1/500 | Santa Cruz Biotechnology | SC-12732 | Corresponding to amino acids 138-158 of rat myogenin | AB_627980 | PMID:19075112 |
| Myosin  Mouse monoclonal | 1/500 | Developmental Studies Hybridoma Bank | MF20 | Chicken myosin sarcomere | AB_2147781 | PMID:23504940 |
| β-tubulin  Mouse monoclonal | 1/5000 | Life Technologies | 480011 | Alpha beta III | AB_10375603 | PMID:12740870 |
| HA  Mouse monoclonal | 1/5000 | Covance Research Products Inc | MMS-101R-500 | HA.11 Clone 16B12 | AB_10063630 | PMID:19075112 |
| FRG1  Mouse monoclonal | 1/500 | Abcam | ab-55024 | Recombinant full-length protein corresponding to amino acids 1-259 of Human FRG1 | AB_941653 | NA |
| FHL1  Goat polyclonal | 1/1000 | Abcam | Ab-23937 | Synthetic peptide corresponding to internal sequence amino acids 261-272 of Human FHL1 | AB_732361 | PMID:24087791 |
| Eid3  Goat polyclonal | 1/500 | Santa Cruz Biotechnology | Sc-167738 | Epitope mapping near the C-terminus of EID-3 of mouse origin | NA | PMID:15987788 |
| Pax7  Mouse monoclonal | 1/50 | Developmental Studies Hybridoma Bank | Pax7 | Chicken paired box gene 7 | AB_528428 | PMID:23784810 |
| MyoD1 (D8G3)XP Rabbit monoclonal | 1/400 | Cell Signaling technology | 13812 | Synthetic peptide corresponding to residues surrounding Gly190 of human MyoD1 protein | NA | NA |
| Dystrophin  Rabbit polyconal | 1/400 | Abcam | Ab-15277 | Synthetic peptide corresponding to amino acids 3661-3677 of Human Dystrophin | AB_301813 | PMID:20886625 |

NA, not available
