# Supplementary material for: FHL1 Reduces Dystrophy in Transgenic Mice Overexpressing FSHD Muscular Dystrophy Region Gene 1 (FRG1)
Source: PLoS One. 2015 Feb 19;10(2):e0117665. doi: 10.1371/journal.pone.0117665 (PMC4335040; doi:10.1371/journal.pone.0117665)
Supplement: S2 Table — (DOC) [file pone.0117665.s007.doc]

**Table S2. Muscle weights from wild type, *FRG1*- and *FRG1/FHL1-* transgenic mice at 6 weeks of age.**

|  | **Wild type**  **n=8** | ***FRG1***  **n=6** | ***FRG1/FHL1***  **n=6** |
| --- | --- | --- | --- |
| Body weight (g) | 26.32 ± 0.24 | 18.72 ± 2.04 | 22.83 ± 2.85 |
| Tibialis Anterior (mg) | 56.25 ± 0.62 | 38 ± 5.67 | 43.58 ± 1.16 |
| Quadriceps (mg) | 212.37 ± 2.77 | 109.50 ± 21.13 | 138.00 ± 2.23 |
| Triceps (mg) | 130.75 ± 3.60 | 73.08 ± 2.75 | 96.16 ± 3.34 |
| Trapezius (mg) | 184.87 ± 6.09 | 110.83 ± 3.06 | 165.16 ± 8.19 |
| Average cumulative muscle weight (mg) | 146.06 ± 4.51 | 82.85 ± 1.69 | 110.72 ± 3.31 |
| Muscle weight relative to  Wild type | 1 | 0.57* | 0.76†‡ |

*p=< 0.0007 and †p=<0.0194 significantly different from wild type; ‡p=<0.0194 significantly different from *FRG1*

Data represent mean ± SEM; n=6-8 per genotype
