# Supplementary material for: FHL1 Reduces Dystrophy in Transgenic Mice Overexpressing FSHD Muscular Dystrophy Region Gene 1 (FRG1)
Source: PLoS One. 2015 Feb 19;10(2):e0117665. doi: 10.1371/journal.pone.0117665 (PMC4335040; doi:10.1371/journal.pone.0117665)
Supplement: S3 Table — (DOC) [file pone.0117665.s008.doc]

**Table S3 Muscle weights from wild type, *FRG1*- and *FRG1/FHL1-* transgenic mice at 12** weeks of age.

|  | **Wild type**  **n=11** | ***FRG1***  **n=12** | ***FRG1/FHL1***  **n=14** |
| --- | --- | --- | --- |
| Body weight (g) | 30.97 ± 0.27 | 21.74 ± 0.20 | 22.41 ± 0.16 |
| Tibialis Anterior (mg) | 65.86 ± 0.78 | 35.87 ± 0.37 | 42.20 ± 0.54 |
| Quadriceps (mg) | 247.63 ± 2.33 | 92.58 ± 1.64 | 105 ± 1.22 |
| Triceps (mg) | 146.5 ± 2.09 | 69.70 ± 0.87 | 87.03 ± 0.83 |
| Trapezius (mg) | 286.20 ± 5.62 | 136.5 ± 3.45 | 160.21 ± 2.22 |
| Average cumulative muscle weight (mg) | 186.55 ± 7.09 | 83.667 ± 10.55 | 98.6125 ± 2.57 |
| Muscle weight relative to Wild type | 1 | 0.46* | 0.55†‡ |

*p=<0.0006 and †p=< 0.0025 significantly different from wild type; ‡p=<0.0088 significantly different from *FRG1;*

Data represent mean ± SEM; n=10-14 per genotype
