# Supplementary material for: FHL1 Reduces Dystrophy in Transgenic Mice Overexpressing FSHD Muscular Dystrophy Region Gene 1 (FRG1)
Source: PLoS One. 2015 Feb 19;10(2):e0117665. doi: 10.1371/journal.pone.0117665 (PMC4335040; doi:10.1371/journal.pone.0117665)
Supplement: S1 Rawdata — (PDF) [file pone.0117665.s009.pdf]

Figure 1A in manuscript

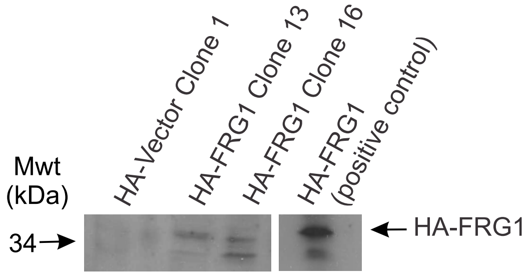

Raw data: FRG1 expression in C2C12-HA-FRG1 myoblasts (clone #13 and #16 were used for further investigation) versus C2C12-HA-vector myoblasts.

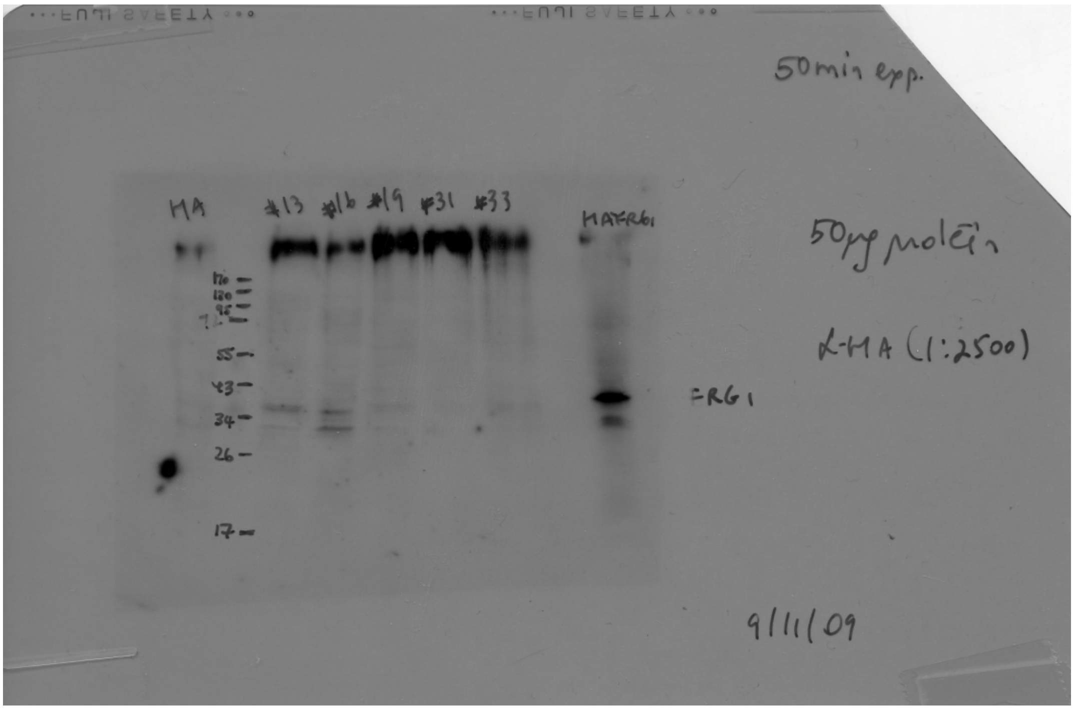

Western blot: HA

Figure 1H in manuscript

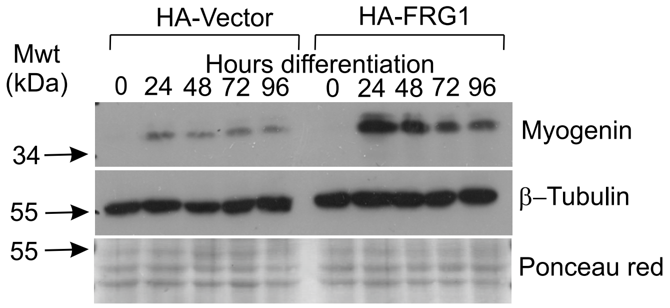

Raw data: myogenin expression in C2C12- HA-vector myoblasts versus C2C12-HA-FRG1 myoblasts

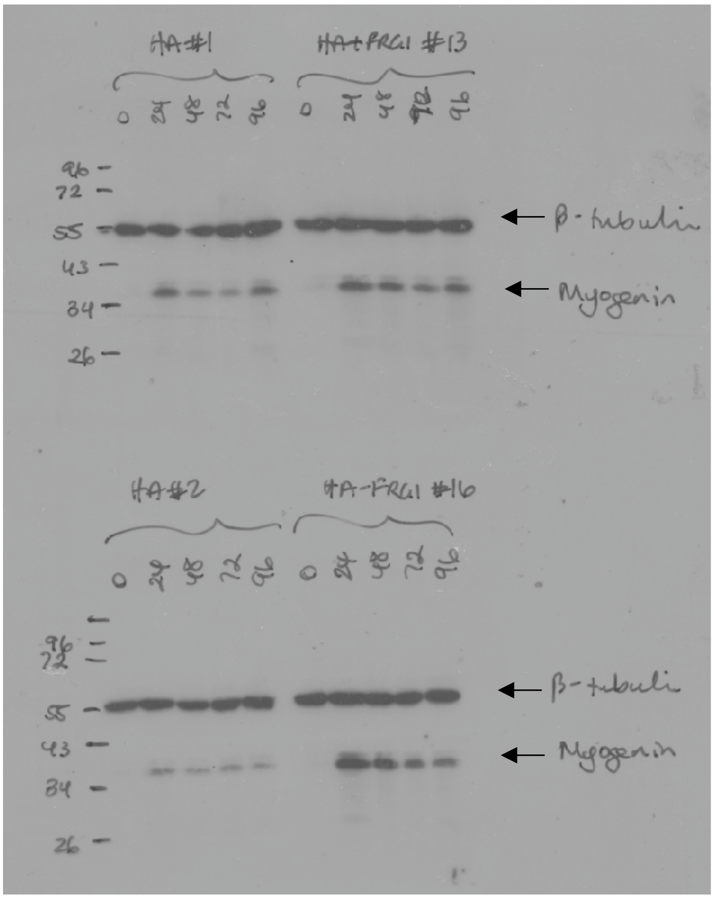

Western blot: myogenin and β-tubulin
Membrane was cut horizontally at 43kD protein marker, and top half probed for β-tubulin and bottom half probed for myogenin

Figure 1J in manuscript

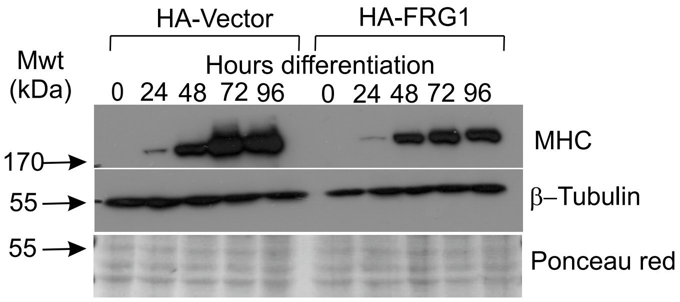

Raw data: dMHC expression in C2C12-HA-vector myoblasts versus C2C12-HA-FRG1myoblasts

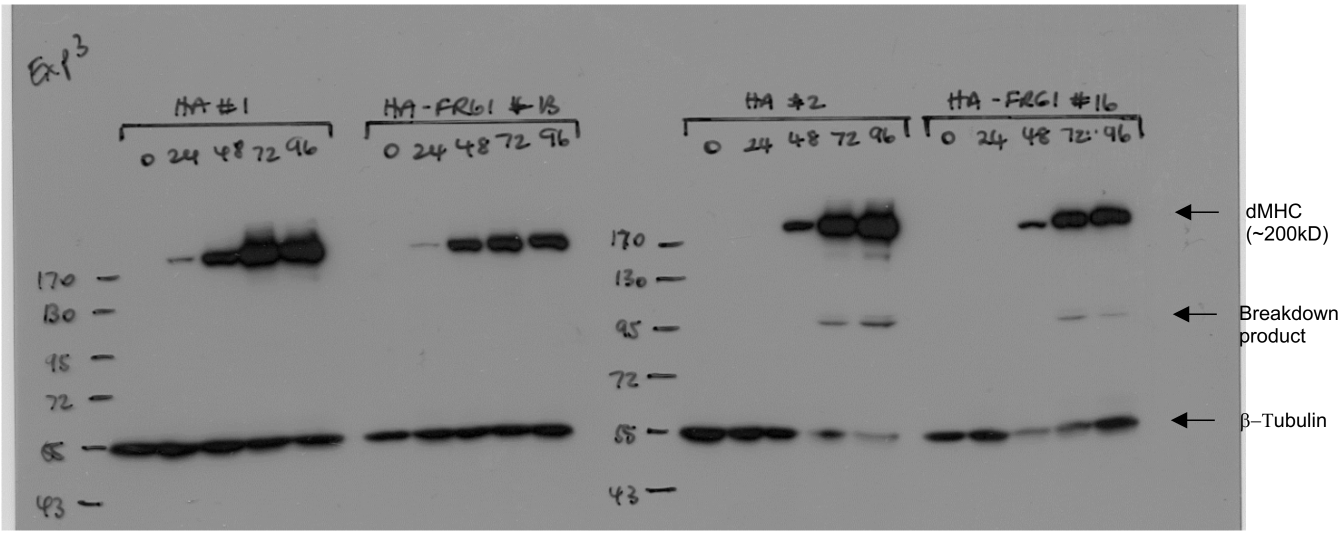

Western blot: dMHC and β-tubulin
Membrane cut horizontally at 72kD protein marker, and top portion probed for dMHC and bottom portion probed for β-tubulin.

Figure 2A in manuscript

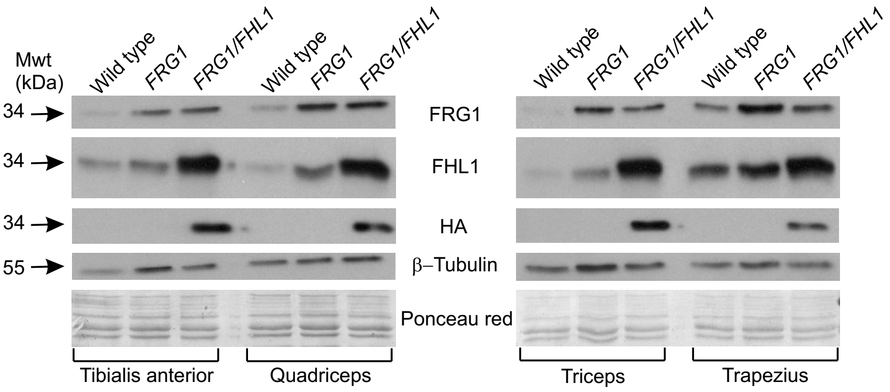

Raw Data - Tibialis anterior and Quadriceps (left panel)

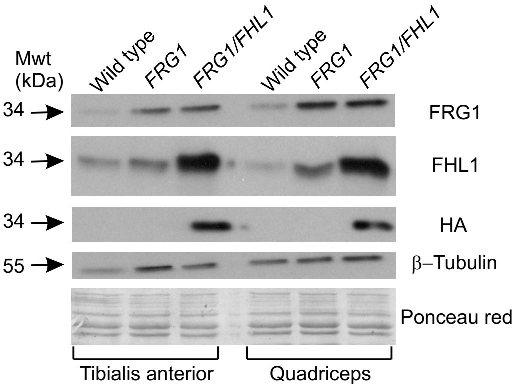

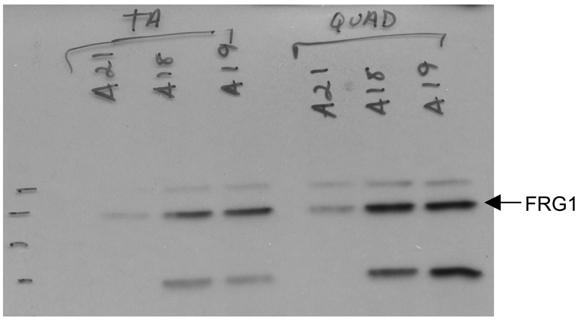

Western blot: FRG1

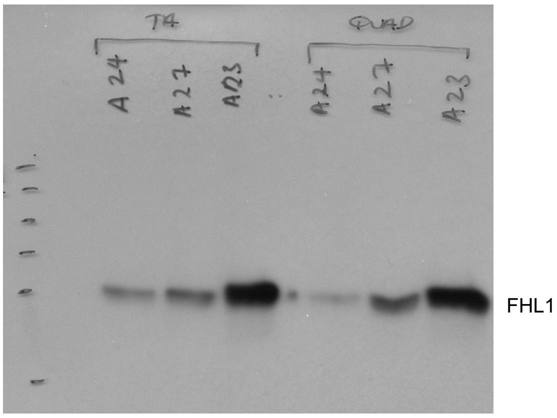

Western blot: FHL1

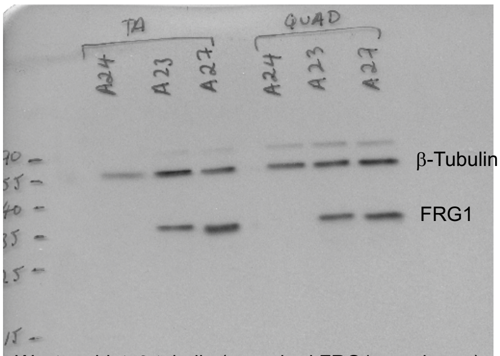

Western blot: β-tubulin (re-probed FRG1 membrane)

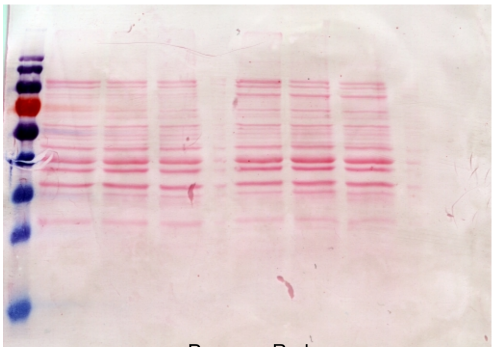

Ponceau Red

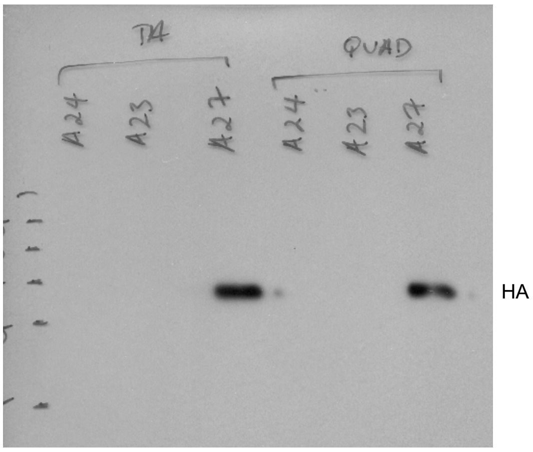

Western blot: HA

Figure 2A in manuscript (Continued)

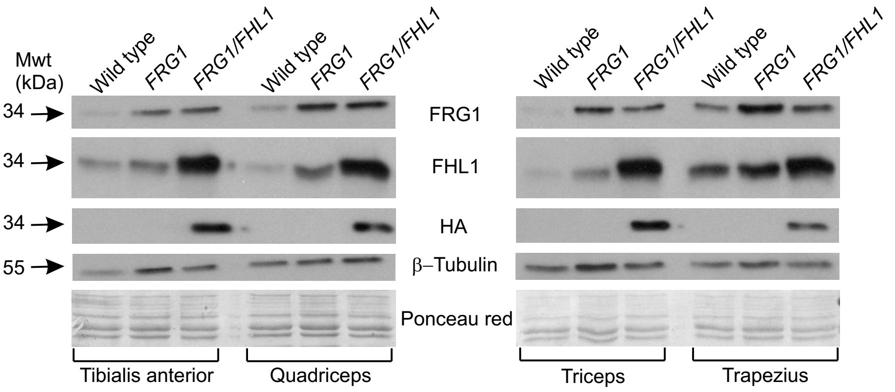

Raw Data - Triceps and Trapezius (right panel)

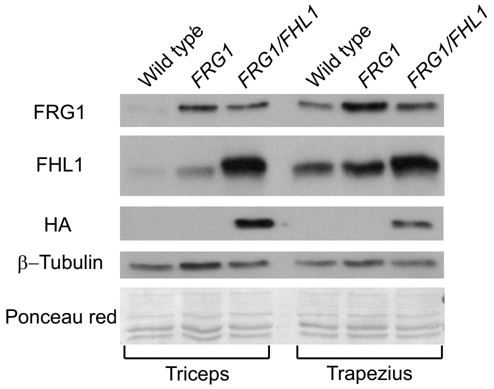

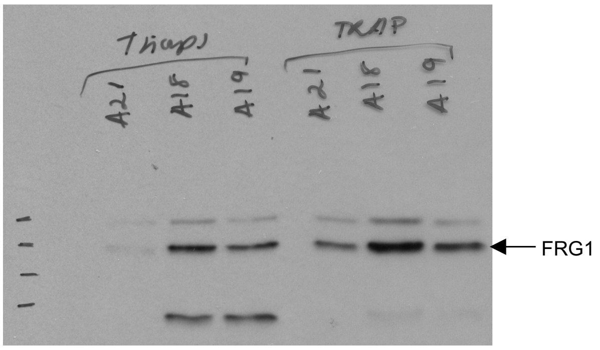

Western blot: FRG1

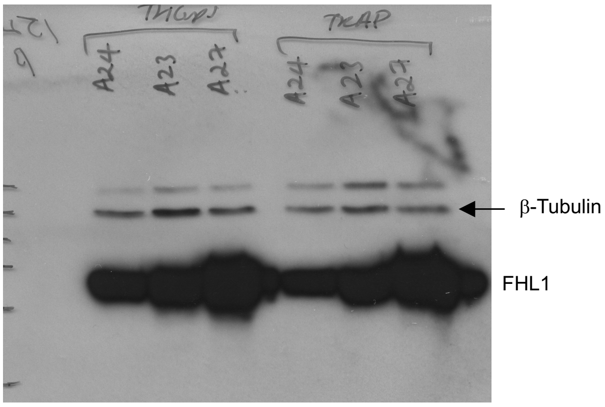

Western blot: β-tubulin (re-probed FHL1 membrane)

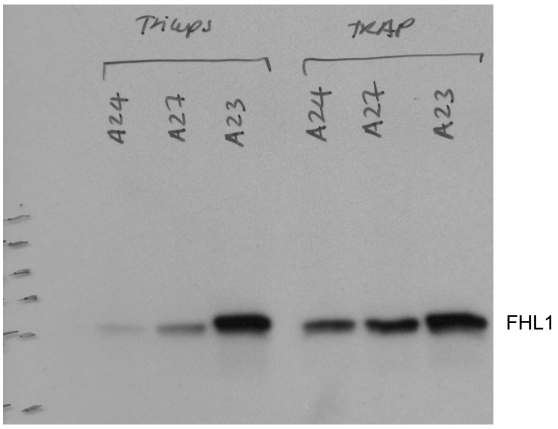

Western blot: FHL1

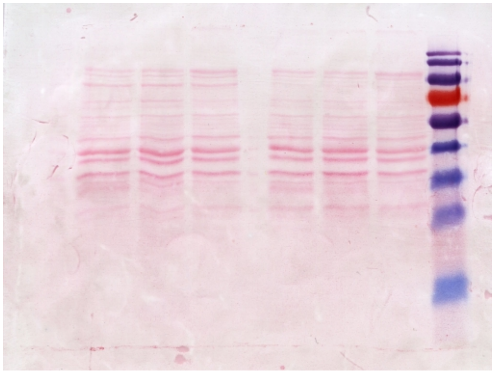

Ponceau Red

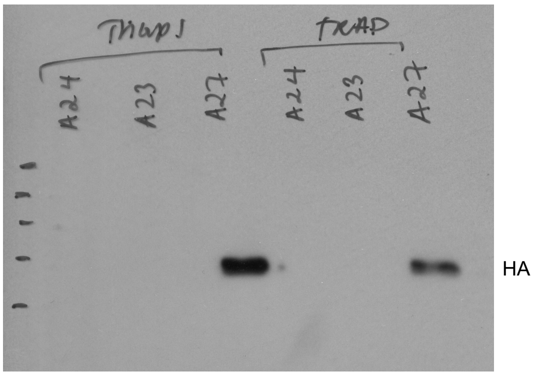

Western blot: HA

Figure S4 in manuscript

Raw Data

I

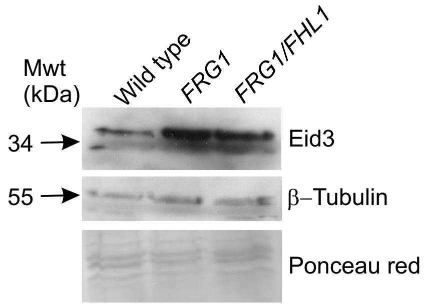

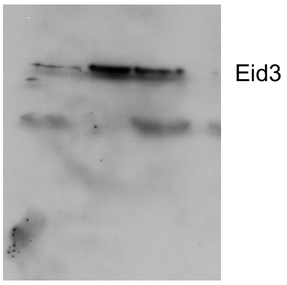

Western blot: Eid3

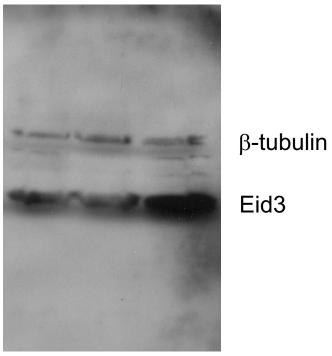

Western blot: β-tubulin (re-probe Eid3 membrane)

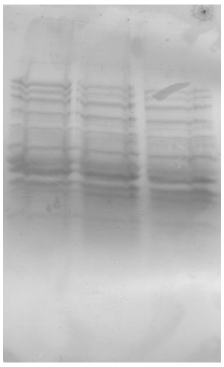

Ponceau Red
